# Influence of GLP-1 on Myocardial Glucose Metabolism in Healthy Men during Normo- or Hypoglycemia

**DOI:** 10.1371/journal.pone.0083758

**Published:** 2014-01-06

**Authors:** Michael Gejl, Susanne Lerche, Annette Mengel, Niels Møller, Bo Martin Bibby, Kamille Smidt, Birgitte Brock, Hanne Søndergaard, Hans Erik Bøtker, Albert Gjedde, Jens Juul Holst, Søren Baarsgaard Hansen, Jørgen Rungby

**Affiliations:** 1 Department of Biomedicine, Aarhus University, Aarhus, Denmark; 2 Department of Endocrinology, Aarhus University Hospital, Aarhus, Denmark; 3 Department of Clinical Biochemistry, Aarhus University Hospital, Aarhus, Denmark; 4 Department of Cardiology, Aarhus University Hospital, Skejby, Aarhus, Denmark; 5 PET Centre, Aarhus University Hospital, Aarhus, Denmark; 6 Department of Neuroscience and Pharmacology, University of Copenhagen, Copenhagen, Denmark; 7 Department of Biomedical Sciences, University of Copenhagen, Copenhagen, Denmark; 8 Department of Biostatistics, Aarhus University, Aarhus, Denmark; 9 Department of Medicine, Viborg Regional Hospital, Viborg, Denmark; 10 Department of Endocrinology, Rigshospitalet, Copenhagen, Denmark; University of Las Palmas de Gran Canaria, Spain

## Abstract

**Background and Aims:**

Glucagon-like peptide-1 (GLP-1) may provide beneficial cardiovascular effects, possibly due to enhanced myocardial energetic efficiency by increasing myocardial glucose uptake (MGU). We assessed the effects of GLP-1 on MGU in healthy subjects during normo- and hypoglycemia.

**Materials and Methods:**

We included eighteen healthy men in two randomized, double-blinded, placebo-controlled cross-over studies. MGU was assessed with GLP-1 or saline infusion during pituitary-pancreatic normo- (plasma glucose (PG): 4.5 mM, n = 10) and hypoglycemic clamps (PG: 3.0 mM, n = 8) by positron emission tomography with ^18^fluoro-deoxy-glucose (^18^F-FDG) as tracer.

**Results:**

In the normoglycemia study mean (± SD) age was 25±3 years, and BMI was 22.6±0.6 kg/m^2^ and in the hypoglycemia study the mean age was 23±2 years with a mean body mass index of 23±2 kg/m^2^. GLP-1 did not change MGU during normoglycemia (mean (+/− SD) 0.15+/−0.04 and 0.16+/−0.03 µmol/g/min, P = 0.46) or during hypoglycemia (0.16+/−0.03 and 0.13+/−0.04 µmol/g/min, P = 0.14). However, the effect of GLP-1 on MGU was negatively correlated to baseline MGU both during normo- and hypoglycemia, (P = 0.006, r^2^ = 0.64 and P = 0.018, r^2^ = 0.64, respectively) and changes in MGU correlated positively with the level of insulin resistance (HOMA 2IR) during hypoglycemia, P = 0.04, r^2^ = 0.54. GLP-1 mediated an increase in circulating glucagon levels at PG levels below 3.5 mM and increased glucose infusion rates during the hypoglycemia study. No differences in other circulating hormones or metabolites were found.

**Conclusions:**

While GLP-1 does not affect overall MGU, GLP-1 induces changes in MGU dependent on baseline MGU such that GLP-1 increases MGU in subjects with low baseline MGU and decreases MGU in subjects with high baseline MGU. GLP-1 preserves MGU during hypoglycemia in insulin resistant subjects.

ClinicalTrials.gov registration numbers: NCT00418288: (hypoglycemia) and NCT00256256: (normoglycemia).

## Introduction

Type 2 diabetes (T2D) is estimated to affect approximately 400 million people before 2030 [1]. The main hormonal and metabolic abnormalities in T2D are dysfunctional insulin secretion, glucagon excess, and insulin resistance [2], eventually beta-cell failure [3] and defects in glucagon-like peptide-1 (GLP-1) secretory responses [4].

Because patients with T2D have elevated circulating free fatty acids (FFA) they also have increased beta oxidation of FFAs at the expense of reduced glucose oxidation. [5]. Compared to glucose oxidation, reliance on fatty acid oxidation for ATP production results in higher costs in mitochondrial oxygen consumption [6], and may become deleterious during myocardial ischemia, which occurs more frequently in diabetic than in non-diabetic subjects due to an increased prevalence of coronary artery disease. Consequently, pharmacological means to switch from free fatty acid to glucose oxidation, when energy demands are challenged, are highly warranted.

The glucagon-like peptide-1 (GLP-1) receptor agonists are validated anti-diabetic medications. They mimic a range of physiological actions of native GLP-1 on glucose metabolism including stimulation of insulin secretion, inhibition of glucagon secretion, inhibition of gastric emptying and reduction of appetite and food intake [7]. Native GLP-1 and GLP-1 analogues are reported to have several significant cardiovascular effects providing the opportunity to address the multifactorial issues involved in increased mortality and morbidity associated with T2D as well as in patients with cardiac disease and no diabetes [8]. The potentially beneficial impact of GLP-1 receptor stimulation may rely on, among others, improved myocardial glucose metabolism [9].

We hypothesized that GLP-1 increases glucose availability and utilization and aimed to assess the effects of GLP-1 receptor (GLP-1R) activation on myocardial glucose uptake (MGU) in healthy subjects with hypo- and normoglycemia.

## Materials and Methods

The protocol for these trials and supporting CONSORT checklist are available as supporting information; see Checklist S1 and Protocol S1, S2, S3, S4.

### Study groups

In the normoglycemia study ten non-smoking, healthy, Caucasian male subjects were included from November 2005 through January 2007. Mean (± SD) age was 25±3 years and BMI was 22.6±0.6 kg/m^2^. In the hypoglycaemia study eight non-smoking, healthy, Caucasian males with a mean age of 23±2 years and a mean body mass index of 23±2 kg/m^2^ participated from January to October 2007. They had a normal physical examination, no history of diabetes or cardiovascular disease, and received no medication. The studies were conducted in accordance with the Declaration of Helsinki and the protocol was approved by the official Regional Science Ethics Committee of Region Midtjylland and County of Aarhus. The participants in both studies were recruited from notices at Aarhus University. All participants received both oral and written information and signed an approved informed consent form before entering the study.

### Study design

**Figure 1:**
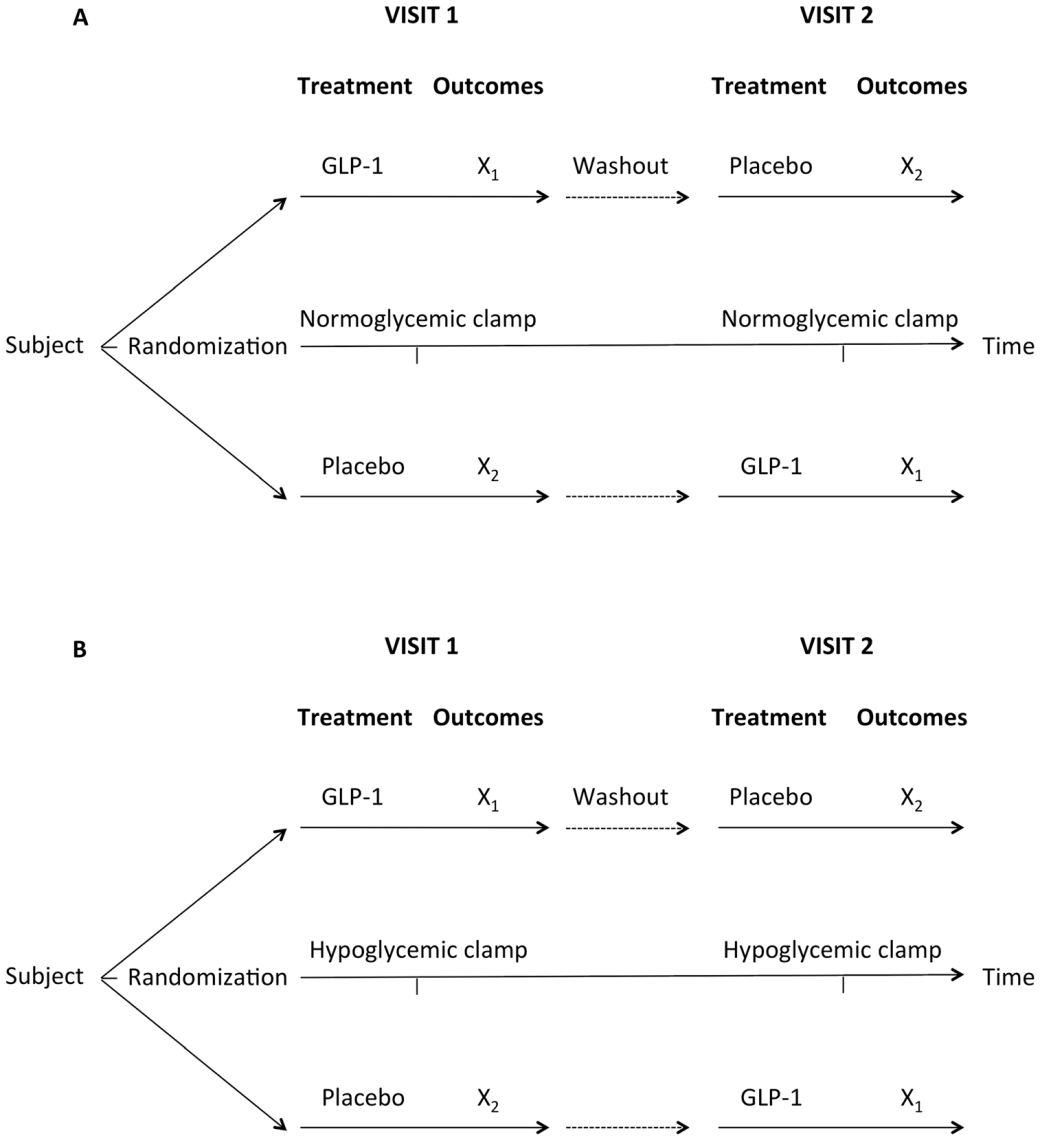
Study design. Design of the normoglycemic study (n = 10) (A) and the hypoglycemic study (n = 8) (B). The studies were conducted as randomized, double-blinded, placebo-controlled, crossover studies. X_1_ and X_2_: PET and clamp data.

The studies were conducted as randomized, double-blinded, placebo-controlled, crossover studies (figure 1 A and B). Each subject was studied twice in random order with GLP-1 and placebo infusion. Positron emission tomography (PET) sessions were separated by an interval of 2–6 weeks. Both sessions commenced at 9.00 hours after an overnight fast. The subjects did not exercise 24 hours prior to the sessions. Subjects were placed in bed and two catheters were inserted for infusion of clamp hormones and for the infusion of GLP-1 or placebo. A third catheter was placed in an arterialized (heated) dorsal hand vein for blood sampling.

An arterial catheter was placed in the radial artery of the right arm in order to draw blood samples for measuring input radioactivity during PET in the hypoglycemia study. In the normoglycemia study a pancreatic-pituitary clamp was performed as described in [10]. In the hypoglycemia study a stepwise hypoglycaemic pancreatic-pituitary clamp was performed according to principles previously described [10], [11]. In brief, somatostatin (Ferring GmbH, D-24109 Kiel) was infused at a rate of 300 µg/hour to suppress the endogenous insulin, glucagon, growth hormone and GLP-1 production. Human glucagon (Glucagen, Novo Nordisk A/S, Copenhagen, Denmark) 0.6 ng/kg/min, growth hormone (Genotropin Miniquik 0.2 mg, Pfizer ApS, Ballerup, Denmark) 2 ng/kg/min were infused with the aim of maintaining near-basal levels (0–360 min). In the normoglycemia study, insulin (Actrapid, Novo Nordisk A/S, Copenhagen, Denmark) was infused at a rate of 0.12 mU/kg/min and in the hypoglycemia study insulin (Actrapid, Novo Nordisk A/S, Copenhagen, Denmark) was infused at a rate of 0.8 mU/kg/min. Glucose (200 g/l) was infused at a variable rates to clamp plasma glucose (PG) at 4.5 mM in the normoglycemic setup and in the hypoglycemia study glucose was infused at variable rates to initially clamp PG at 4.5 mM (0–150 minutes). From 150 to 180 minutes, PG was lowered to 4.0 mM and maintained at this level until 210 minutes. From 210 minutes to 240 min PG was lowered to 3.5 mM and maintained at this level until 270 minutes. From 270 min to 300 min PG was lowered to a nadir of 3.0 mM and maintained at this level during PET (360–400 min). GLP-1 or placebo infusion was initiated at time 60 minutes and maintained during the entire session.

The subjects received either intravenous synthetic GLP-1 (7–36 amide) or placebo at a rate of 1.2 pmol/kg/min (60–360 min) [12]. Recombinant human GLP-1 (7–36 amide) was a kind gift from BioNebraska Inc., Lincoln, NE (USA) and was tested and found positive for sterility and negative for bacterial endotoxins before use. It was dissolved in a sterile buffer containing 600 mg of acetic acid, 50.7 g of mannitol and sterile water added up to 1000 g and had a pH of 4.5. The concentration of GLP-1(7–36amide) was 1 mg/ml and vials of 0.25 ml were stored at −20**°** Celsius. The test solution consisted of 0.25 ml GLP-1, 20 ml of human albumin “ZLB” 5% and sodium chloride (9 g/l) to yield 100 ml of solution. The placebo solution consisted of the above mentioned buffer solution containing human albumin and saline. One subject experienced transient nausea and vomiting during hypoglycemia and GLP-1 infusion with onset at 20 min after initiation of the infusion, lasting approximately 1½ hour. Another subject experienced headache during placebo infusion after 2 hours of infusion, lasting 1 hour.

PG was measured in duplicate every 10 min until PG reached 3.0 mM and then every fifth minute. In the normoglycemia study, PG was measured every 10 min. Blood for measuring insulin, c-peptide, glucagon, GLP-1 (total and intact), growth hormone, FFA, cortisol and ghrelin was drawn every 30 min. Blood for measuring epinephrine and norepinephrine was drawn at 0, 150 and 240 min and every 30 min during the PET scan. Arterial blood for measuring input radioactivity was drawn at predetermined intervals during PET (12×5 s, 8×30 s, 8×300 s and 4×600 s).

### Assays

Plasma glucose was measured immediately after sampling on a Beckman glucose analyzer (Beckman, Palo Alto, CA). All other blood samples were stored at −20°C (C-peptide at −80°C) until assay. Serum NEFAs were analyzed by a commercial kit (Wako Chemicals, Neuss, Germany). Serum GH was analyzed using chemiluminescence technology (IDS-iSYS Multi-Discipline Automated Analyzer; Immunodiagnostic Systems Nordic, Herlev, Denmark). Serum insulin and C-peptide was analyzed using time-resolved fluoroimmunoassay assay (AutoDELFIA PerkinElmer, Turku, Finland). Serum cortisol was measured using a DRG ELISA kit (DRG Instruments, Marburg, Germany). Serum ghrelin (total levels) was measured in duplicate by an in-house assay [13]. Plasma catecholamines were measured by liquid chromatography [14]. Plasma glucagon was measured by radioimmunoassay [15]. All coefficients of variation (inter- and intra-assay) were <9.5%. The assay for intact GLP-1 is an enzyme-linked immunosorbent assay using unextracted plasma, which was collected and stored in the presence of a dipeptidyl peptidase-IV inhibitor (valine-pyrrolidide, 0.01 mmol/l, final concentration added to the blood sample immediately after collection). Intra- and inter-assay coefficient of variations are 2 and 5%, respectively [16]. Total GLP-1 was analyzed using a C-terminal radioimmunoassay for amidated GLP-1. Intra- and inter-assay coefficients of variation for total GLP-1 are <6% and <15%, respectively [17].

### Positron emission tomography

A PET scanner model EXACT HR47 (Siemens Medical, Knoxville, USA) with a 15 cm axial field of view and an acquisition capacity of 47 simultaneous transaxial planes with a spatial resolution of 4–5 mm at the center of the field of view was used. All PET data were acquired in 2D mode. After a 1 min scout scan for patient positioning, we did a correction for photon attenuation, based on a 15 min. transmission scan, using ^68^Ge rod sources acquired prior to the ^18^F-fluordeoxyglucose (^18^F-FDG) scans.

### Myocardial glucose uptake (MGU)

MGU was quantified by PET using ^18^F-FDG as metabolic tracer. ^18^F-FDG was injected (200 MBq diluted in 10 ml saline) and a dynamic emission scan consisting of 3 frames (3×10 min) was acquired from 60 min after tracer injection (brain scans were acquired from min 0–45). The software used for region of interest allocation and analysis was Carimas (Turku PET centre, Turku, Finland). MGU was quantified by fitting tissue and blood pool (from arterial blood samples in the hypoglycemia study and from image-derived arterial inputs in the normoglycemia study) time activity curves to a linearization Gjedde-Patlak model for ^18^F-FDG [18], [19]. Using this model, we obtained the value of net clearance for ^18^F-FDG (K). For 4 subjects in the hypoglycemia study, in which arterial blood samples were not collected during the heart scan, the missing points in the input curve were estimated by bi-exponential extrapolation from eight samples collected during the brain scan 10–45 min after tracer injection. In the normoglycemia study, image-derived arterial input functions were automatically obtained by localizing the carotid artery on the PET images for each subject from each study day as described in [20] and the missing points were collected from images derived from the left ventricle during heart scan. The lumped constant is the conversion factor between the net uptakes of ^18^F-FDG and glucose which depend on the unidirectional clearances and the phosphorylation rates. In this study, we used a fixed lumped constant on 1. We used iFit (www.liver.dk/ifit.html) for kinetic modelling. MGU was calculated as: MGU = PG_*_K/ρ_m_, where PG is plasma glucose during scan, K is clearance of ^18^F-FDG and ρ_m_ is myocardial density.

### Insulin sensitivity

Insulin resistance homeostasis model of assessment 2 (HOMA 2IR) was computed as described in ref. [21].

### Calculations and statistical analysis

Primary outcome was MGU.

The PET data were analyzed using a paired student's t-test. The plasma-data were analysed using a linear mixed effects model with subject and all interactions involving subject, including the interaction between subject and time and the interaction between subject and treatment, as random effects. Treatment (GLP-1 vs. placebo), time, and the interaction between the two were included in the analysis as fixed effects. A linear regression model was used for regression analysis of K and MGU and Pearson's r test was used to evaluate K, MGU and insulin resistance (HOMA2 IR). The statistical software used was GraphPadPrism (GraphPad Software) and Stata (StataCorp LP, College Station, Texas, USA) with a significance level of 5%. Data are presented as mean ± SD. The study was planned as preliminary exploration and thus the number of subjects that entered the study did not derive from a power calculation.

## Results

### PET

#### Normoglycemia study (figure 2)

**Figure 2:**
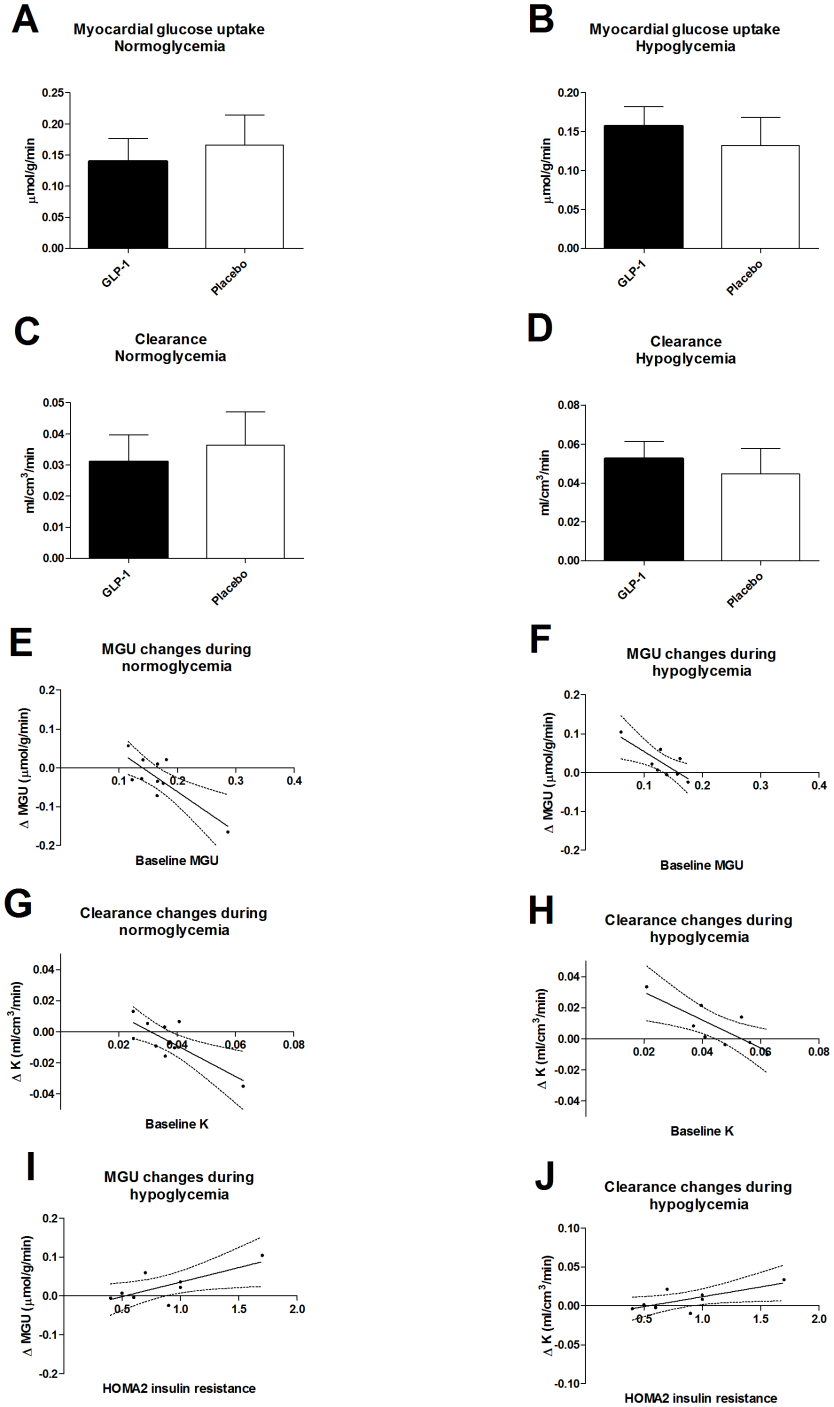
Positron emission tomography. Myocardial glucose uptake (MGU) during normoglycemia and hypoglycemia (A and B). ^18^F-FDG clearance (K) during normoglycemia and hypoglycemia (C and D). Relation between placebo MGU and change in MGU during GLP-1 infusion in the normoglycemia study (E), placebo MGU and change in MGU during GLP-1 infusion in the hypoglycemia study (F). Relation between placebo K and change in K during GLP-1 infusion in the normoglycemia study (G), placebo K and change in K during GLP-1 infusion in the hypoglycemia study (H). HOMA 2IR and the change of MGU during GLP-1 infusion in the hypoglycemia study (I). HOMA 2IR and the change of K during GLP-1 infusion in the hypoglycemia study (J). Data are mean ± SD. Regression lines with 95% confidence intervals.

GLP-1 did not affect MGU (placebo: 0.17±0.05 and GLP-1: 0.14±0.04 µmol/g/min, P = 0.21) (figure 2A) nor K (placebo: 0.04±0.01 and GLP-1: 0.03±0.01 ml/cm^3^/min, P = 0.25) (figure 2C). Regression analysis showed a linear relationship (P = 0.009, r^2^ = 0.59) between the baseline (placebo) K and the alteration in K after GLP-1 infusion (figure 2G). GLP-1 increased K of ^18^F-FDG and glucose of cardiomyocytes in subjects with low baseline K suggesting decreased K in subjects with high baseline K. Correspondingly, there was a linear relationship between the baseline MGU and the GLP-1-induced alterations in MGU (P = 0.006, r^2^ = 0.64) (figure 2E), an enhanced response was found with low baseline MGU and a decreased response with high baseline MGU.

#### Hypoglycemia study (figure 2)

GLP-1 did not affect MGU (placebo: 0.13±0.04 and GLP-1: 0.16±0.03 µmol/g/min, P = 0.14) (figure 2B) nor K (placebo: 0.045±0.01 and GLP-1: 0.053±0.009 ml/cm^3^/min, P = 0.17) (figure 2 D). Regression analysis showed a linear relationship (P = 0.016, r^2^ = 0.65) between the baseline (placebo) K and the alteration in K after GLP-1 infusion (figure 2H). GLP-1 increased K of ^18^F-FDG and glucose of cardiomyocytes in subjects with low baseline K suggesting decreased K in subjects with high baseline K. Correspondingly, there was a linear relationship between the baseline MGU and the GLP-1-induced alterations in MGU (P = 0.018, r^2^ = 0.64) (figure 2F), a higher response was found with low baseline MGU and a decreased response with high baseline MGU. The alterations of K and MGU correlated positively with HOMA 2IR (P = 0.046 and 0.037, r^2^ = 0.51 and 0.54) (figure 2I and 2J).

### Hormones and metabolites

#### Normoglycemia study (figure 3)

**Figure 3:**
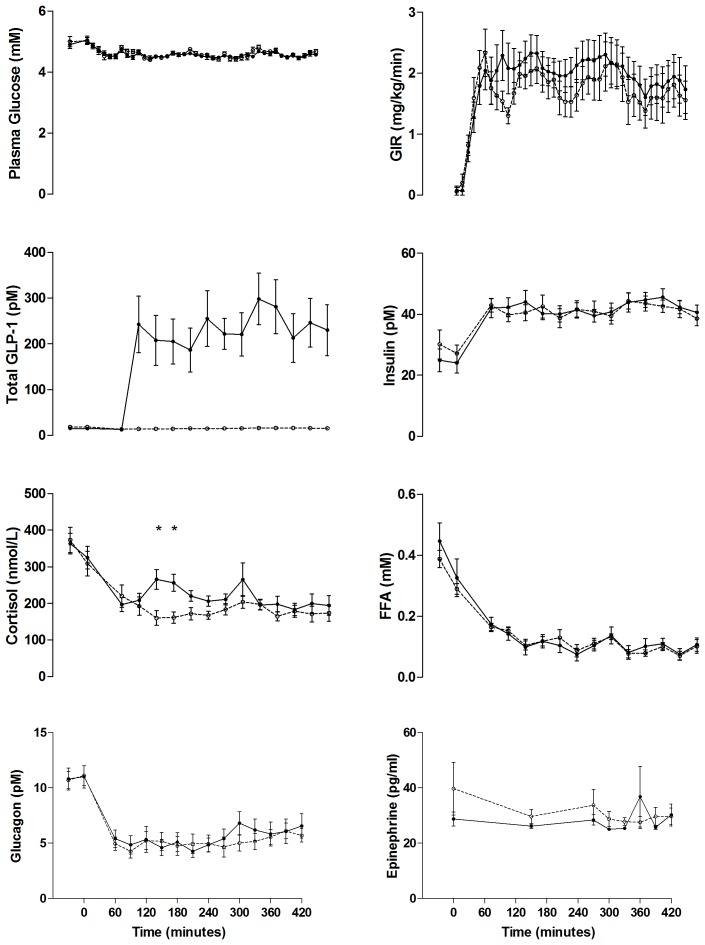
Hormones and metabolites - normoglycemia study. Plasma glucose, glucose infusion rates (GIR), total GLP-1, insulin, cortisol, free fatty acid (FFA), glucagon and epinephrine concentrations during GLP-1 (black dots) and placebo infusion (white dots). Data are means ± SEM. * P≤0.05.

GLP-1infusion increased circulating GLP-1 concentrations as shown in figure 3. Concentrations differed between study days regarding intact and total GLP-1, both P<0.001. PG and glucose infusion rates were the same with placebo and GLP-1 (all P>0.4). The plasma C-peptide and FFA levels were suppressed and no differences were found between placebo and GLP-1 (P = 0.58 and 0.74). There were no significant differences in circulating growth hormone, insulin or glucagon (P = 0.51, 0.99 and 0.36) between sessions. Ghrelin (data not shown, p = 0.26), norepinephrine (data not shown, p = 0.20), and epinephrine levels were also similar during sessions, p = 0.39. Serum cortisol concentrations were significantly higher with GLP-1 at 120 and 150 min (P<0.001), but similar during PET scans, P>0.28.

#### Hypoglycemia study (figure 4)

GLP-1 infusion increased circulating GLP-1 concentrations. Concentrations differed between study days regarding intact and total GLP-1, both P<0.001. During the PET scan, the PG concentration remained at 3.15±0.09 (GLP-1) and 3.14±0.16 (placebo) mM, *P* = 0.67. The glucose infusion rate increased with GLP-1 infusion, P = 0.01. The plasma C-peptide and FFA levels were suppressed and no differences were found between groups (P = 0.73 and 0.67). There were no significant differences in circulating growth hormone (P = 0.14) between sessions. Glucagon increased in the GLP-1 group during clamp step PG<3.5 mM (time 270, 330, 360 and 420 min.), P<0.05. Counter-regulatory hormones rose as expected when PG declined. Insulin levels were similar between sessions, *P* = 0.52. Ghrelin (data not shown, p = 0.30), norepinephrine (data not shown, p = 0.75), and epinephrine levels were also similar during sessions, p = 0.98. Serum cortisol concentrations were significantly higher with GLP-1 at PG steps of 4.5 and 4.0 mM, P = 0.0001–0.04 but were similar at PG steps of 3.5 and 3.0 mM., P = 0.86, GLP-1 vs. placebo.

## Discussion

GLP-1 did not change overall MGU or K during normo- or hypoglycemia. The GLP-1 conditioned MGU is negatively correlated to baseline MGU such that GLP-1 increased MGU in subjects with low baseline MGU and decreased MGU in subjects with high baseline MGU during both normo- and hypoglycemia. This alteration correlated with the grade of insulin resistance of the subjects in the hypoglycemia setup. Moreover, the glucose infusion rate was increased with GLP-1 despite increased glucagon secretion during hypoglycemia.

**Figure 4:**
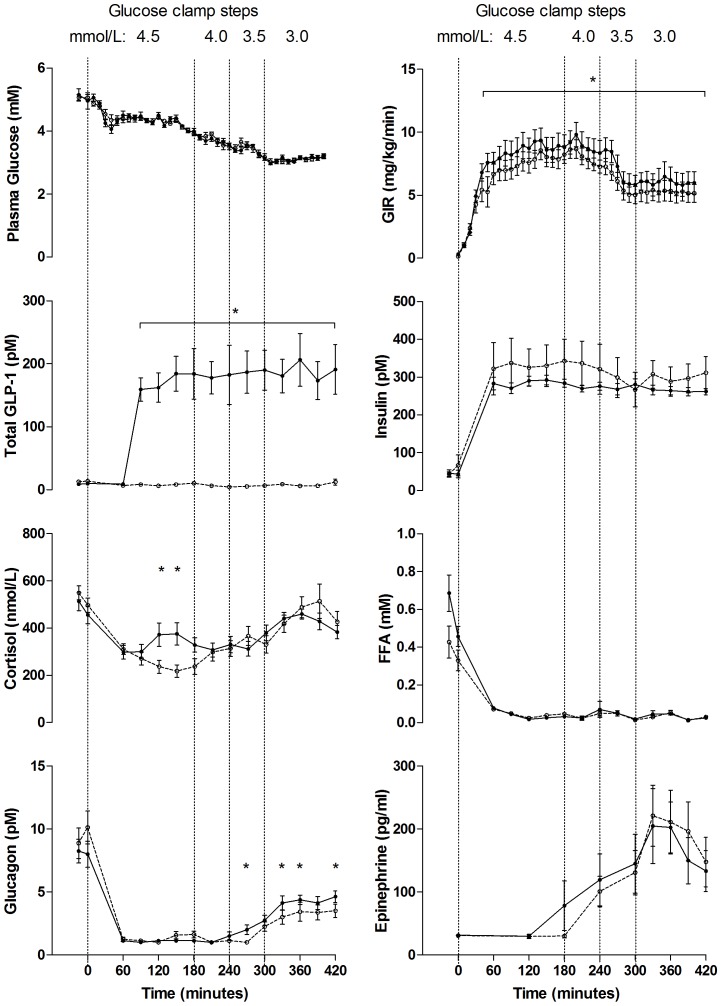
Hormones and metabolites - hypoglycemia study. Plasma glucose, glucose infusion rates (GIR), total GLP-1, insulin, cortisol, free fatty acid (FFA), glucagon and epinephrine concentrations during GLP-1 (black dots) and placebo infusion (white dots). Data are means ± SEM. * P≤0.05.

In a healthy heart, the substrate preferences are flexible. In normal conditions a minimum of 60% of ATP is derived from the oxidation of free fatty acids, and the rest from the oxidation of glucose. In the diabetic heart, ATP is predominantly derived from myocardial fatty acid oxidation [5]. This has been associated with increased myocardial oxygen consumption and decreased mechanical efficiency [22]. During hypoxia, energy metabolism shifts toward a greater oxidation of glucose to maintain myocardial viability, as the oxidation of glucose consumes less oxygen than oxidation of free fatty acids. Clinical and experimental studies have shown that increased glucose uptake during acute myocardial ischemia is associated with preserved cardiac function [23], [24]. Hyperglycaemia is an independent predictor of cardiovascular risk, infarct sizes are reported to be directly related to the severity of hyperglycaemia and the glycometabolic state is associated with the mortality risk in T2D patients with acute myocardial infarction [5]. The beneficial effect of lowering blood glucose to very low levels, however, is controversial, one of the major problem being the increase in the number of hypoglycemic events [25]. However, the studies also showed that the currently available anti-diabetic treatments fail to reach acceptable glycemic control in the majority of patients. Therefore treatment strategies with beneficial effects toward reducing macrovascular complications (e.g., stroke, myocardial infarction) and with a low risk of adverse hypoglycemic events are being tested and although hypoglycemic events are reported in patients with heart failure [26], [27], GLP-1 analogues are considered agents with low risk of hypoglycaemia. GLP-1 inhibits glucagon secretion from the pancreatic α-cells at fasting and elevated glucose concentrations, whereas hypoglycemia induced glucagon secretion is not inhibited - the mechanisms are not fully understood [28]. GLP-1 infusion resulted in pharmacologically relevant plasma concentrations of the intact hormone in both studies [12]. Slightly higher glucagon levels during hypoglycemia in the GLP-1 group compared to the control experiments were previously found in studies with native GLP-1 [29], with the GLP-1 analogue exenatide [10] and the the DPP-IV inhibitor vildagliptin [30]. We found an increased secretion of glucagon during hypoglycemia despite somatostatin infusion with GLP-1 infusion and furthermore glucose infusion rates increased in the hypoglycemia study with GLP-1. Together the latter indicate a possible mechanism for alleviation of hypoglycemia by GLP-1. In the PET studies, increased levels of cortisol were observed with GLP-1 infusion during the clamp from time 120–150 min after which cortisol levels were similar (figure 3 and 4). This increase in cortisol is consistent with previous observations [31], [32] and may indicate an activation of hypothalamic neuroendocrine neurons by GLP-1 [33]. Central administration of GLP-1 activates the hypothalamo-pituitary-adrenocortical axis primarily through stimulation of corticotropin releasing hormone neurons [34]. Myocardial glucose uptake is not correlated to cortisol levels as reported in [35], and cortisol might even decrease MGU primarily by stimulating lipolysis and by elevating FFA concentration. However; cortisol levels were similar 1 hour prior to and during the PET scans and therefore the observed effects of GLP-1 on myocardial observed are most likely not influenced by cortisol.

GLP-1 exerts its actions through the GLP-1R. The GLP-1R may be present in cells of many tissues including the vascular endothelium, cardiomyocytes, endocardium and smooth muscle cells. Some cardiac effects of GLP-1R agonists may be exerted via other receptors [36]. Prior studies have demonstrated the presence of GLP-1R in the myocardium [36], [37], but recent papers [38]–[40] have questioned the interpretation of data obtained using standard antisera to detect the authentic GLP-1R.

Growing evidence, reviewed by Chinda K. et al [41], demonstrate cardioprotective effects of GLP-1 and GLP-1 analogues during ischemia-reperfusion in both animal and clinical models despite inconsistent reports. The exact mechanisms have never been fully elucidated, although many mechanisms have been proposed (e.g. increased MGU, reduction of oxidative stress and proapoptotic kinase, activation of prosurvival kinase, and attenuation proinflammatory cell activation) [41]. The GLP-1R agonists reduce ischemia-reperfusion injury by reducing infarct size and improve left ventricle function [41], and long term studies show that patients treated with the GLP-1 agonist exenatide are less likely to record a CVD event [42]. However, the impact of these so-called cardioprotective effects of GLP-1 is still less well established. The most comprehensive meta-analysis of the GLP-1 analogues did not find any significant GLP-1 effect on CVD events as a whole [43]. Therefore, although several possible mechanisms have been put forward suggesting that GLP-1s could have a protective effect on the cardiovascular risk profile (e.g., reductions of blood glucose, body weight, and blood pressure, improvement in left ventricular ejection fraction, changes in cardiac metabolism, lipid metabolism, arthrosclerosis development and endothelial function and the response for ischemia-reperfusion injury [44]), reasons for a (possible) decreased risk of developing CVD are still poorly understood. Interestingly, Kim et al [39] demonstrated that the receptor is localized predominantly in the cardiac atria and that GLP-1 stimulates the secretion of atrial natriuretic peptide (ANP). In rat neonatal cardiac myocytes, hypoxia significantly increased glucose uptake stimulated by ANP, but ANP did not affect basal glucose uptake under normoxic conditions [45] as examined in the present study and in a similar study of patients with T2D [11] where no direct effect on MGU are demonstrated despite a 24% increase in myocardial blood flow. The T2D study revealed that the acute stimulation of GLP-1R did alter the myocardial glucose metabolism dependent on the baseline myocardial glucose metabolism and hence level of insulin resistance [11], consistent with the findings in the present study. A recent paper reports an increase in MGU in healthy lean subjects with GLP-1 infusion [46]. The study differs from the present studies by not having clamped the participants and consequently not having completely comparable PG, insulin, and FFA between the groups as well as non-suppressed FFA. Furthermore, they infused a GLP-1 at a higher rate (1.5 pmol/kg/min).Hypoglycemia is associated with increased mortality rates in diabetic patients after ischemia and reperfusion and the underlying mechanisms may involve reduced capacity for preconditioning. As the salvage of the myocardium is associated with increased MGU during reperfusion, cardioprotection is thought to be linked to glucose metabolism [47]. An important mechanism of the beneficial cardiovascular effect of GLP-1 may be the increase of MGU in the subjects with the lowest baseline MGU – the most insulin resistant subjects, regardless of glycemia. Glucose mainly enters myocardial cells via the facilitative glucose transporters, GLUT1 and GLUT4. As most of the glucose transported across the GLUT1 and GLUT4 into the myocardium is metabolized due to high hexokinase affinity, our results support that transport across the cell membranes is affected by acute infusion of GLP-1 as indicated in [11]. The regression estimates indicate that GLP-1 raises GLUT translocation in subjects with low baseline GLUT activity and lowers the activity in subjects with high baseline GLUT activity. Thus, it seems that the baseline activity of glucose transporters and hence level of insulin resistance influences the GLP-1 mediated action of K and MGU in cardiomyocytes. Furthermore, the effects of GLP-1 on K and MGU regress positively with HOMA2 IR (figure 2I and J). This is in accordance with observations in models of T2D where reduced translocation of GLUT1 and GLUT4 is found in the heart [48] and, the GLUT1 and GLUT4 protein levels in the membrane are related to fasting glucose levels, the higher the fasting glucose are, the lower glut concentration in the membranes of the cardiomyocytes [49]. Fasting glucose and IR are highly correlated. Growing evidence [37], [50], [51] indicates that GLP-1 and analogues enhance GLUT1 translocation in myocardial cell membranes and GLUT4 protein levels of the myocardium, but no reports support hexokinase activity to be directly influenced by GLP-1 in contrast to glucokinase activity in β-cell lines [52].

Although the mechanism is not clear, it is speculated that signaling through the GLP-1R facilitates activation of Akt or AMP-activated protein kinase (AMPK) and consequently GLUT4 translocation [50]. Alternatively that stimulation of translocation of GLUT1 and GLUT4 is mediated by increased myocardial NO production [53], [54] - facilitated by activation of the GLP-1R [37]. GLUT1 is reported to be controlled partly by levels of plasma glucose and GLP-1 in other tissues, e.g. in the blood brain barrier in various cerebral regions [29], [55]. The mechanism of the dual effect of GLP-1 and the analogue exenatide remains unclear but may be caused by receptor-mediated changes or may be a general feature of intracellular IR. The duality may be beneficial because glucose transport and uptake are stabilized by GLP-1R activation in subjects with low insulin sensitivity.

The practical implications of these findings are most importantly that GLP-1 preserves myocardial glucose metabolism during hypoglycemia in insulin resistant subjects.

Limitations: In the intact human organism, the cardiac lumped constant varies with the metabolic condition [56], [57]. Lumped constant was predefined to 1. This may underestimate the MGU, but the underestimations are the same in the groups due to the cross-over design of the study and as the subjects were clamped in both settings. GLP-1 and the analogue exenatide do not seem to affect lumped constant in the preceding brain scan or in previous myocardial ^18^F-FDG scans [11], [29], [55]. Comparisons of absolute MGU between the normoglycemia and the hypoglycemia state could be compromised by the different methods used for determination of the input curve. This was not the case for comparisons between GLP-1 and placebo within the normo- or hypoglycemia groups.

In conclusion GLP-1 does not enhance MGU overall. GLP-1 increases MGU in subjects with low baseline MGU and decreases MGU in subjects with high baseline MGU. During hypoglycemia the most insulin resistant subjects increased their MGU. The GLP-1 induced increased secretion of glucagon and glucose infusion rates during hypoglycemia despite somatostatin indicate a possible alleviation of hypoglycemia by GLP-1. Studies addressing MGU in both the hypoxic state (under possible influence of ANP) and long-term studies are needed to provide more profound knowledge regarding the potential beneficial impact of GLP-1R stimulation on MGU.

## Supporting Information

Protocol S1 **Normoglycemia.**
(DOCX)

Protocol S2 **Hypoglycemia.**
(DOCX)

Protocol S3 (DOC)

Protocol S4 (DOC)

Checklist S1 CONSORT checklist.
(DOC)
