# Supplementary material for: Influence of GLP-1 on Myocardial Glucose Metabolism in Healthy Men during Normo- or Hypoglycemia
Source: PLoS One. 2014 Jan 6;9(1):e83758. doi: 10.1371/journal.pone.0083758 (PMC3882300; doi:10.1371/journal.pone.0083758)
Supplement: Protocol S2 — Hypoglycemia. (DOCX) [file pone.0083758.s002.docx]

**The Effect of GLP-1 on Glucose Uptake in the Brain and Heart in Healthy Subjects During Hypoglycemia Assessed by Positron Emission Tomography**

Type 2 diabetes mellitus, T2D is a disease characterized by an immense growing prevalence world wide. T2D is associated with a three-fold increase in cardiovascular complications (myocardial infarction and stroke) leading to significantly higher morbidity and mortality in this group of patients. The prospective British Diabetes Study (UKPDS) showed that neither diet alone nor the pharmaceutical treatment utilized (Sulphonylurea, Metformin, Insulin) were able to reduce these macrovascular complications. GLP-1 (glucagon-like-peptide-1)is an incretin with convincing effects on glycaemia in type 2 diabetic patients with little or no risk of hypoglycaemia. New research in animal models has shown a potential protective effect in the brain and heart in association with ischaemic damage. The mechanism behind this protective effect is not known. During hypoglycaemia the brain lacks glucose which is the main fuel for sufficient brain function. The brain will compensate by increasing glucose uptake across the blood brain barrier and similarly in the heart.

The effect of native GLP-1 on glucose uptake in the brain and heart will by visualized by fluoro-deoxy-glucose FDG-PET-scan during hypoglycaemia in healthy men. At the same time a pancreatic/pituitary clamp will be performed. The hypothesis is that GLP-1 directly will stimulate glucose uptake independent of the pancreatic hormones and through this mechanism exert neuro- and cardioprotective actions.

Comparisons: FDG-uptake in the brain and heart with GLP-1 infusion compared to placebo.

Study type: Interventional

Design: Allocation: Randomized

Endpoint Classification: Pharmacokinetics/Dynamics Study

Intervention Model: Crossover Assignment

Masking: Double Blind (Subject, Caregiver, Investigator, Outcomes Assessor)

Primary Purpose: treatment

- Intervention:
- Drug: **glucagon-like-peptide-1** intravenous infusion of 1.2pmol/kg/min for 7 hours
- Drug: placebo intravenous infusion of 1.2pmol/kg/min
- Study arms: Active Comparator: A Intervention: Drug: **glucagon-like-peptide-1**
- Placebo Comparator: P Intervention: Drug: placebo

Inclusion Criteria:

- Healthy men
- Age 20-50 years
- Caucasian
- BMI 20-30 kg/m2

Exclusion Criteria:

- Diabetes in subject and 1.degree relatives

Any disease of clinical relevance
