# Supplementary material for: Influence of GLP-1 on Myocardial Glucose Metabolism in Healthy Men during Normo- or Hypoglycemia
Source: PLoS One. 2014 Jan 6;9(1):e83758. doi: 10.1371/journal.pone.0083758 (PMC3882300; doi:10.1371/journal.pone.0083758)
Supplement: Protocol S4 — (DOC) [file pone.0083758.s004.doc]

# PROTOKOL

**Effekten af GLP-1 på glucoseoptagelsen i CNS og hjerte hos raske personer under *hypoglycæmi* vurderet ved Positron Emissions Tomografi.**

*Susanne Lerche, læge og klinisk assistent (Projektkoordinator)*

*Ole E. Schmitz, professor, dr.med (Klinisk ansvarlig investigator)*

*Albert Gjedde, professor, dr.med (PET ansvarlig)*

***PhD-vejledere***

*Ole E. Schmitz, professor, dr. med*

*Albert Gjedde, professor,dr.med*

*Birgitte Brock, overlæge,PhD*

*Farmakologisk institut, Århus Universitet,*

*Forskningsenheden, Med. af M og*

*PET-centeret*

*Århus Sygehus NBG*

*8000 Århus C*

*Juni 2006*

1. **Baggrund og hypotese 3**
2. **Formål med studiet 4**
3. **Design 4**
4. **Deltagerudvælgelse 4**

*Rekruttering 4*

*Inklusionskriterier 5*

*Eksklusionskriterier 5*

*Tilbagetrækningskriterier 5*

1. **Hormoner og dosering 5**
2. **Metoder 6**

*Pancreatisk clamp undersøgelse 6*

*PET-scanningr 6*

*Laboratorieanalyser 7*

1. **Undersøgelsesdesign og flowchart 7**
2. **Statistik 8**
3. **Ulemper, risici og bivirkninger 8**

*Ulemper og risici 8*

*Strålehygiejne 9*

*Medicinbivirkninger 9*

1. **Etiske aspekter og informeret samtykke 9**
2. **Strålingdosis og samfundsmæssig nytteværdi 10**
3. **Databehandling 11**
4. **Økonomiske forhold 11**
5. **Tidsperspektiv 11**
6. **Publikationspolitik 11**
7. **Forsøgsansvarlig og studiegruppe 11**

**Referenceliste 12**

**Samtykkeerklæring 14**

**Information til forsøgspersoner 15**

**Lægmandsbeskrivelse 19**

**Fuldmagt 21**

**Opslag til rekruttering af forsøgspersoner 22**

**Appendix**

*Flow-charts*

*Brev fra Lægemiddelstyrelsen vedr. GLP-1*

**1. Baggrund og hypotese**

Type 2 diabetes T2D er stigende i incidens både i Danmark og i størstedelen af den vestlige verden. T2D ledsages hyppigt af multiple komplikationer, hvor især de makrovaskulære komplikationer i form af iskæmisk hjertesygdom og cerebrale infarkter er ansvarlige for en forøget morbiditet og mortalitet hos denne patientgruppe. Der er således en 3-fold øgning i disse kardiovaskulære komplikationer hos type 2 diabetespatienten i forhold til baggrundsbefolkningen. (*MTV rapport 2003*).

Det er vist, både for type 1 diabetes(DCCT) og type 2 diabetes(UKPDS), at en forbedring af den glycæmiske kontrol nedsætter risikoen for mikrovaskulære (øjne, nyrer, nerver) komplikationer. Det samme er imidlertid **ikke** tilfældet for de makrovaskulære komplikationer.(1,2)

Det er imidlertid velkendt at optimal glycæmisk kontrol med insulin ofte begrænses af en forøget risiko for **hypoglycæmi.** Særligt hos de diabetespatienter der har mistet evnen til føling samt evnen til at etablere et tilstrækkeligt modregulatorisk respons (mange type 1 diabetikere og patienter med type 2 diabetes gennem mange år) kan dette få alvorlige cerebrale følger.

Glucose er nemlig det primære brændstof for hjernen. Under faste sker der en fordeling af organismens glucoseomsætning således, at først og fremmest CNS tilføres tilstrækkelig substrat. 50 % af glucoseforbruget foregår således i hjernen(insulin-uafhængig) under faste, 25 % i lever og mavetarmkanal(insulin-uafhængig) samt 25 % i muskelvæv inkl. hjerte og i fedtvæv (insulin-afhængig) (3).

Hypoglycæmi er således en cerebral stress situation, hvor der ikke tilføres tilstrækkelig glucose til at opretholde normal nervecelle-funktion. Der sker derfor iværksættelse af flere kompensationsmekanismer. Hypoglycæmien registreres initialt især via centrale ”glucose-sensorer”. Disse er lokaliseret til den ventromediale del af hypothalamus.

På denne måde startes iværksættelsen af det modregulatoriske respons (primært adrenalin og glucagon), som skal genoprette normalt glucoseniveau både perifert og centralt.(4)

Der kompenseres ligeledes gennem forøget glucoseoptagelse (via faciliteret transport) over blodhjernebarrieren, samt en minimering af glucoseforbruget i hjernen for om muligt at opretholde normal cerebral metabolisme og derved beskyttelse af nervecellerne.(5)

Glucagon-like-peptid-1 (GLP-1) er et inkretint hormon med talrige dokumenterede effekter på det glycæmiske respons. Det er et af de mest potente insulinotropiske hormoner kendt og frigives fra L-celler i tarmens mucosa som respons på fødeindtagelse. Effekter på Ø-cellerne er glucoseafhængig stimulation af insulinsekretionen, hvor der kræves tilstedeværelse af glucose for at GLP-1 kan udøve sin virkning. Samtidig potentieres den insulinotrope effekt af glucose kraftigt, men insulinsensitiviteten påvirkes ikke på kort sigt. Der ses en hæmning af glucagon sekretionen. GLP-1 har hæmmende virkning på ventrikeltømning og appetit medieret via påvirkning af nerveceller i hypothalamus.(6,6)

I dyre- og cellulære forsøg stimuleres β-celle neogenese, vækst og differentiering, og in vitro ses hæmning af β-celle apoptose(7)*.* GLP-1 virker således også som en β-celle vækstfaktor.

GLP-1 behandling har indtil videre vist sig **ikke** at medføre hypoglycæmi.(7)

I forbindelse med hypoglycæmi og samtidig GLP-1 tilførsel er tidligere vist et bevaret modregulatorisk respons(8,9).

Således er GLP-1-analoger særdeles attraktive til behandling af type 2 diabetes.

GLP-1 receptorer(GLP-1R) findes på α-, og β-cellerne i pancreas, men findes derudover også i flere andre organer. Receptorerne i CNS og i hjertet er fundet identiske med den pancreatiske receptor. GLP-1 har således en sandsynlig væsentlig effekt i disse organer.(10) I hjernen er GLP-1R især repræsenteret i hypothalamus, men findes også i de øvrige hjerneafsnit.

Talrige receptorer findes i hjertet.

Ved binding af GLP-1 til receptoren stimuleres enzymet adenylatcyklase med deraf følgende stigning i intracellulær cAMP førende til aktivering af forskellige enzymsystemer(MAP-kinase, PI3-kinase). Aktivering af enzymet PI3Kinase og også stimuli der øger cAMP-dannelsen er vist at udøve beskyttelse af celler i hjertet og i hjerne.(11,12)

GLP-1 har vist sig at have flere potentielle *ekstrapancreatiske* effekter, deriblandt flere beskyttende effekter.(13)

- I 3 nylige studier er GLP-1 vist at have både **neuroprotektive** og **neurotrofiske** egenskaber(14,15,15,16)
- Nikolaidis et al har i et nyligt studie vist, at GLP-1 har **cardioprotektive** egenskaber(17,18)).
- To studier har påvist en beskyttende effekt af GLP-1 i hjertet **u**afhængigt af insulin(18,19)

*Idet GLP-1 i henhold til ovenstående har potentiel beskyttende effekt i CNS og hjerte er vores* ***hypotese****, at GLP-1 ved hypoglycæmi vil stimulere glucoseoptagelsen i hjernen og i hjertet* ***u****afhængigt af insulin og glucagon i forhold til placebo og derved udøve en direkte beskyttende effekt på cellerne i hjerne og hjerte under hypoglycæmi.*

*Vi vil undersøge dette med* ***ny metodologi,*** *hvor der ses bort fra GLP-1’s påvirkninger af insulin og glucagon. Således ses udelukkende på GLP-1’s* ***egen virkning.***

*Vi mener, at studiet på dette grundlag vil give væsentlig information om en mulig virkningsmekanisme bag en potentiel protektiv effekt af GLP-1 i hjernen og i hjertet under hypoglycæmi.*

*Studiet foretages i forlængelse af tilsvarende forsøg fortaget under normoglycæmi.*

**2. Formål med studiet**

Med ny metodologi at undersøge effekten af nativt GLP-1 på glucoseoptagelsen i CNS og hjerte vurderet ved optagelse af 18-fluro-deoxy-glucose(FDG) og samtidig PET-scanning af raske mandlige forsøgspersoner under en **trinvis hypoglycæmisk pancreatic clamp**.

- At sammenligne effekten af GLP- 1 på den non-insulinstimulerede glucose optagelse i CNS med placebo hos raske mænd ved hypoglycæmi.
- At sammenligne effekten af GLP-1 på den non-insulinstimulerede glucose optagelse i Hjertet med placebo hos raske mænd ved hypoglycæmi.

**3. Design**

Randomiseret, dobbelt-blindet cross-over-design. Hver forsøgsdeltager vil blive undersøgt 2 gange i tilfældig rækkefølge henholdsvis med GLP-infusion og med placebo.

Der vil være et ca. 4 ugers interval mellem de 2 undersøgelsesdage.

Der vil blive etableret en *trinvis hypoglycæmisk pancreatisk clamp.* Den pancreatiske clamp udføres i lighed med *Nielsen et al* i 2004 (20) kombineret med trinvis hypoglycæmisk clamp som foretaget af *Degn et al*(8).

PET scanning foretages af hjerne og hjerte med FDG(18-fluoro-deoxy-glucose) som tracer og vil blive foretaget ved p-glucose (PG)-nadir på 3,0mM.

Randomiseringskonvolutter vil blive fremstillet af kollega uden deltagelse eller interesse i projektet.

**4. Deltager udvælgelse**

10 raske mænd mellem 20 og 50 år skal fuldføre studiet. Det forventes at randomisere 10-15 personer.

Kvinder inkluderes ikke idet deres insulinfølsomhed varierer med menstruationscyklus.

Rekruttering: Der vil blive lavet opslag på diverse institutter på Århus Universitet.(Opslag er vedlagt)

Inklusionskriterier:

- Informeret samtykke før studierelateret aktivitet
- Mænd
- Alder > 20 år og < 50 år
- BMI > 20 kg/m2 og < 30 kg/m2
- BT< 140/90
- Kaukasere

Eksklusionskriterier

- Diabetes hos personen selv eller hos 1.grads slægtninge (inkluderer nedsat glucosetolerance)
- Klinisk betydende lever eller nyreinsufficiens (s-ALAT > 2 gange øvre reference eller s-creatinin > 130mM)
- Klinisk betydende anæmi
- Anden klinisk relevant abnorm biokemisk værdi
- Aktuel eller tidligere tilstedeværelse af èn af følgende sygdomme med klinisk relevans:
  1. hjertekarsygdom
  2. leversygdom
  3. nyresygdom
  4. lungesygdom
  5. G-I lidelse
  6. endokrinologisk sygdom
  7. CNS-lidelse
  8. hæmatologisk lidelse
  9. tab af 100ml blod eller mere indenfor de sidste 2 mdr
  10. manglende compliance
  11. kendt misbrug af alkohol eller medicin
  12. rygning
  13. deltagelse i klinisk studie mindre end 3 mdr før inklusion i dette studie
  14. kendt allergi overfor studiehormoner
  15. indtagelse af medikamenter/naturmedicin med mulig indvirken på sukkerstofskiftet
- Personer, der indenfor en periode på 1 år har deltaget i videnskabelige undersøgelser, hvor der er brugt isotoper, eller som har fået foretaget større diagnostiske undersøgelser, hvor der er anvendt ioniserende stråling.

Tilbagetrækningskriterier:

- Tilbagetrækkelse af informeret samtykke
- Uacceptabel compliance
- Alvorlig bivirkning
- Bivirkning som efter investigators mening nødvendiggør, at deltageren udgår af studiet.

**5. Hormoner og dosering**

GLP-1 er et naturligt forekommende peptid hormon, som er kendetegnet ved at have en særdeles kort halveringstid og derfor **ikke har potentiale som et lægemiddel**(Der henvises til vedlagte brev fra Lægemiddelstyrelsen vedrørende **GLP som værktøj** i forbindelse med nyligt godkendt projekt). Det er tidligere anvendt i flere studier. De øvrige hormoner som anvendes i den pancreatiske clamp anvendes her ligeledes som værktøjer i forsøgsdesignet og ikke som lægemidler.

Idet GLP-1 ikke kan betragtes som lægemiddel anmeldes studiet ikke til Lægemiddelstyrelsen.

Der anvendes følgende doseringer:(20).

- Somatostatin (Ferring) 60ng/kg/min
- Insulin actrapid (NOVO Nordisk) 0,8mU/kg/minut
- Glucagon (NOVO Nordisk,Glucagen) 0,6ng/kg/minut
- Væksthormon (NOVO Nordisk) 2ng/kg/minut
- GLP-1 1,2pmol/kg/min*.*(22)

**6. Metoder**

Pancreatisk og trinvis hypoglycæmisk clamp

Somatostatin indgives for at hæmme den endogene produktion af insulin, glucagon og væksthormon. Derudover hæmmer somatostatin også den endogene produktion af GLP-1.

Insulin, glucagon og væksthormon gives for at opretholde basale hormon- niveauer.

Der etableres en trinvis hypoglycæmisk clamp, hvor PG ved hjælp af varierende glucoseinfusioner fastholdes på henholdsvis 4,5mM i de første 150min af studiet, herefter sænkes PG til 4,0mM over 30min og fastholdes i dette niveau i 30min, herefter sænkes PG til 3,5mM over 30min og fastholdes her i alt 30min for herefter at sænkes til nadir på 3,0mM over 30min og fastholdes i dette niveau i max 2 timer under samtidig PET-scanning af CNS og hjerte.

Ved PG<4,5 vil GLP-1 ikke stimulere insulinsekretion ej heller hæmme glucagonsekretionen..(8,9)

PET-scanning

Udføres på PET-centeret, Århus Sygehus, NBG i henhold til afdelingens vanlige retningslinier. Der anlægges venflon i kubitalvene til indgift af det radioaktivt mærkede sporstof ^18^F-fluoro-desoxy-glukose(FDG).

Ved PET-scanningen bliver forsøgspersonen placeret på scannerlejet med hovedet/brystkassen i scanneren. Scanningen foregår under samtidig *trinvis hypoglycæmisk pancreatic clamp*, som beskrevet ovenfor.

**Scanningsforløb:**

1. trin: I forbindelse med hver undersøgelse optages der først et 10min.(hjerne)/20 min.(hjerte) varende **attenuationsscan,** hvor en radioaktiv kilde føres rundt om personen for at måle den specifikke reduktion af strålingen for den enkelte person.

2.trin: Et **metabolisk scan** til bestemmelse af hjernens og hjertets glukoseoptagelse ved hjælp af sporstoffet ^18^F-fluoro-desoxy-glukose (FDG), som indgives intravenøst i en mængde på 200 MBq opløst i fysiologisk saltvand. Sporstoffet gives umiddelbart før scanning over ca. 1 minut.

**Samlet scanningstid vil være ca. 2 timer.**

Der foretages kvantitative vurderinger af glukoseoptagelsen. Billedbehandlingen foregår ved hjælp af 2D acquisition, som begrænser behovet for radioaktiv tracer indgift.

Under scanningsproceduren udtages fra en arterialiseret(opvarmet) venflon blodprøver som anført i studiedesignet.

Hjernes og hjertets glucoseoptagelse beregnes ud fra PET-scanningsbilleder og tracerkinetiske studier af FDG-optagelsen. Den såkaldte ”lumped constant” er korrektionsfaktoren i omregningen af cerebral/kardiel FDG-optagelse til glucose-optagelse. Konstanten påvirkes af ændringer i hjernens/hjertets metabolisme og af ændringer i glucoseniveau.

I dette studie vil vi anvende PET og en tracerkinetisk model som tillader bestemmelse af individuelle lumped konstants og præcise bestemmelser af glucoseoptagelse ud fra analyse af FDG-optagelsen (21,23)

Der anvendes ligeledes en autoradiografisk metode til beregning af glucosestofskiftet i både hjerne og hjerte ud fra én enkelt FDG-injektion.

I forbindelse med PET undersøgelsen anlægges en arteriekanyle på forsøgspersonerne mhp. at udtage blod til måling af specifik radioaktivitet i arterieblodet. Målingen skal anvendes i den senere analyse af PET-scanningsbillederne.

Arteriekanylen anlægges i a. radialis af en speciallæge i anæstesi i henhold til gældende retningslinier.

Laboratorieanalyser: Laboratorieanalyserne udføres på Medicinsk Forskningslaboratorium, Århus Sygehus samt Centrallaboratoriet, Århus Sygehus.

- Der gemmes serum og plasma til eventuel senere analyse af andre metabolitter. Der er **ikke** tale om en Biobank, derimod analyse af markør for oxidativt stress. Analysemetoden kendes endnu ikke - såfremt analysen bliver umuligt, vil blodet blive destrueret. Det gemte blod vil ikke blive anvendt til andet end ovennævnte.

MR-scanning

Der indføres en besøgsdag mere, hvor der vil blive foretaget en **anatomisk MR-scanning af hjernen** mhp. at få en ”skabelon” af den enkelte forsøgspersons hjerne. Skabelonen skal anvendes i analysefasen af PET-scanningsbillederne.

MR-scanningen foretages på Århus Universitetshospital og varer ca. 5-10 min.

**7. Undersøgelsesdesign og flow-chart**

**1.besøg = screening:**

- Foregår på medicinsk afd. M, Århus Sygehus NBG, varer ca. ½-1 time.
- Inklusions- og eksklusionskriterier samt informeret samtykke
- Generel objektiv undersøgelse inkl. BT, højde og vægt.
- Blodprøver: PG, hæmoglobin, Leukocytter +diff.tælling, CRP, trombocytter, Creatinin, carbamid, albumin, ALAT, bilirubin, ASAT, basisk fosfatase, totalt calcium, natrium og kalium.

Blodprøverne ved screeningsbesøget analyseres på Centrallaboratoriet på Århus Sygehus.

Screeningsbesøget kan finde sted op til 4 uger før besøg 2.

**2. og 3. besøg = Clamp + PET undersøgelse (4 uger imellem besøgene)**

- Foregår på PET- centeret Århus Sygehus NBG, besøgene varer ca. 8-10 timer pr gang.
- Forsøgspersonen møder kl. 7.30 og har fastet siden kl. 22 dagen inden (gerne drikke vand)
- Der anlægges 2 intravenøse adgange i albuebøjningerne samt en arterialiseret (opvarmet ved hjælp af varmekasse over hånden) venflon i den ene håndryg til blodprøvetagning. Der tages blodprøver til t= -30min (p-glucose, C-peptid, insulin, GLP-1, glucagon) og til t=0 (p-glucose, insulin, C-peptid, glucagon, GLP-1, katekolaminer, cortisol, GH, Ghrelin, FFA).

Herefter opstartes den pancreatiske clamp undersøgelse således:

0 min: Somatostatin, insulin, væksthormon og glucgon opstartes i doseringer som anført ovenfor.

PG clampes derefter til 4,5mM ved hjælp af varierende infusion af 20 % glucose.

Koncentrationen af insulin, væksthormon, glucagon og somatostatin vil blive holdt konstant under undersøgelsen.

I løbet af 1 time bringes clampen i steadystate med PG på 4,5mM.

60 min: GLP-1 infusion (1,0 pmol/kg/min) eller placebo (isoton nacl) intravenøst startes.

150-180 min: PG sænkes til 4,0mM

180-210 min: PG fastholdes på 4,0mM

210-240 min: PG sænkes til 3,5mM

240-270 min: PG fastholdes på 3,5mM

Til tiden 260min lejres forsøgspersonen i PET-scanneren.

270-300 min: PG sænkes til 3,0mM

300-420 min: PG fastholdes på **nadir 3,0mM** og samtidig foretages PET scanning

PET-scanning: Til tiden 295min indgives tracer i form af FDG 200MBq opløst i saltvand og umiddelbart herefter til T=300min startes PET scanning af hjernen og hjertet som beskrevet.

**Blodprøver:**

- PG måles hvert 5.-10.min under hele clampen samt ved behov. Der vil blive foretaget dobbeltmålinger af PG.
- Insulin, glucagon, GH, GLP-1, C-peptid, katekolaminer, cortisol, ghrelin og FFA måles i henhold til ”flow-chart”.

Efter endt PET-scanning stoppes infusionerne, dog fortsætter glucoseinfusionen indtil PG er sikkert stigende. Forsøgspersonen gives et let måltid og PG kontrolleres ca. hver 15. minut indtil det er stabilt ≥ 5mM. Personen udstyres med druesukker samt informeres grundigt om symptomerne på hypoglycæmi.

Herefter afsluttes forsøget.

2. forsøgsdag forgår på akkurat samme måde og der vil være ca. 4 ugers interval mellem forsøgsdagene.

**4. besøgsdag**

Der foretages MR-scanning som beskrevet ovenfor. Besøgsdagen varer ca. ½ time.

Foregår i MR-scanneren på Århus Universitetshospital.

Et ”**flow-chart”** af undersøgelsen findes i Appendix 1og 2.

**8. Statistik**

Til sammenligning inden for grupperne anvendes en parret T-test eller tilsvarende non-parametrisk test. Til sammenligning af de 2 grupper indbyrdes anvendes uparret T-test eller tilsvarende non-parametrisk test.

P-værdier under 0,05 vil blive antaget for statistisk signifikante.

Beregning af antal forsøgspersoner: I tidligere FDG/PET-studie foretaget af Bøtker et al (21) anvendtes lignende design, som i vores studie. Heri var 8 forsøgspersoner tilstrækkelig til den statistiske analyse, idet man her anvendte et parret design.

Derfor vurderes det, at 10 personer er tilstrækkeligt i vores studie.

Kun data fra forsøgsdeltagere, som har fuldført hele studiet vil indgå i den statistiske analyse. Studiet er et ”per protokol” studie, hvor udgåede forsøgsdeltagere vil blive erstattet af nye forsøgsdeltagere. Studiet afsluttes således først når 10 forsøgsdeltagere har fuldført hele studiet.

**9. Ulemper, risici og bivirkninger**

**Ulemper og risici**

Ved anlæggelse af venflon til blodprøvetagning og infusioner samt tracerindgift, er der ganske lille risiko for overfladisk betændelse i huden.

I forbindelse med den pancreatiske clamp undersøgelse bliver der anlagt venflons i begge albuebøjninger. Blodprøver vil blive udtaget fra en venflon anlagt i blodåre på håndryggen.

Pancreatisk clamping har været foretaget flere gange på afd. M uden opståede komplikationer. Hypoglycæmisk clamp er ligeledes foretaget tidl. i afd. M.

Ved PET scanning anbringes forsøgsdeltageren på scannerlejet med hovedet/brystkassen i scanneråbningen. Denne er kun ca. 60cm dyb, således at forsøgspersonen ved hovedscanning har resten af kroppen udenfor scanneråbningen. Der understøttes med puder, således at personen ligger behageligt. Det er vigtigt at personen ligger helt stille under scanningen. Forsøgspersonerne må ikke sove under undersøgelsen.

Der er ingen lydafgivelse fra scanneren fraset en svag blæselyd.

Personen kan under hele undersøgelsen komme i kontakt med personalet deriblandt den ansvarlige læge, der foretager undersøgelsen.

Den totale mængde blod der udtages til blodprøve-analyser under undersøgelsen vil være maximalt 200ml. Samtidig vil der være tid til at regenerere det tabte blodvolumen i tiden mellem de 2 undersøgelses-dage.(ca. 4 uger)

I forbindelse med anlæggelse af arteriekanylen kan der i meget sjældne tilfælde ses følgende komplikationer: blødning, infektion, karpaltunnel syndrom, sene eller muskelskader samt perifer iskæmi og gangræn pga. tromber, embolier og spasmer.

Forsøgspersonen vil grundigt blive informeret om disse risici samtidig med, at det også er indført i deltagerinformationen.

I forbindelse med MR-scanningen kan der opstå minimalt ubehag i form af klaustrofobi samt høje lyde fra scanneren.

MR-scanning anses for at være en sikker undersøgelse uden risici.

**Strålehygiejne**

**Attenuations scan**

Strålebelastning

Dag 1 og dag 2, hjerne og hjerte. 0,1mSv x 4 ca.0,4mSv

**Metabolisk scan**

^18^F-fluorodesoxyglukose giver ifølge ICRP publikation 80

en strålebelastning på 0.019mSv per MBq.

Der gives i dette projekt 200 MBq på 2 forskellige dage

svt en strålebelastning på 2 x 200MBq x 0.019mSv ca.7,6mSv

Strålebelastning i alt ca.8,0mSv

Dette svarer til ca. 3 gange den baggrundsbestråling (3mSv) alle mennesker i Danmark udsættes for på 1 år. Man kan beregne at stråledosis ved dette forsøg med 2 PET scanninger svarer til en ekstra risiko for at dø af kræft på ca. 0,04 %.

Den samlede risiko for at dø af kræft på længere sigt øges herved fra 25 % til 25,04 %.

**Medicinbivirkninger**

GLP-1 har været anvendt i talrige studier og bivirkninger rapporteret er gastrointestinale i form af let kvalme, abdominalt ubehag samt evt. opkastninger, som især optræder ved høje fysiologiske doseringer. Tilsvarende dosering som anvendt i dette studie er anvendt i flere andre studier uden at foranledige gastrointestinale bivirkninger i form af kvalme(24).

Somatostatin infusion kan give kvalme, opkastninger og mavesmerter.

Det er sandsynligt, at forsøgspersonerne vil opleve symptomer på den iværksatte hypoglycæmi under studiet. De vil blive grundig informeret om dette inden forsøgets start.

Alvorlige hændelser og bivirkninger (medførende død, livstrussel, hospitalsindlæggelse eller vedvarende invaliditet), som skønnes relateret til behandlingen vil øjeblikkeligt blive indberettet til den lokale Videnskabsetiske komité i Århus Amt.

Ansvaret for indberetning af bivirkninger påhviler projektgruppen. Enhver hændelse eller bivirkning vil blive behandlet efter gældende standard og vil blive fulgt indtil hændelsen er forsvundet eller indtil tilstanden har stabiliseret sig.

**10. Etiske aspekter**

GLP-1 har som beskrevet potentielle beskyttende effekter i både hjerne og hjerte samtidig med en veldokumenteret effekt på det glycæmiske respons. Type 2 diabetes præges af en markant overhyppighed af iskæmisk hjertesygdom og cerebrovaskulære infarkter, som det med de nuværende behandlingsprincipper ikke har været muligt at nedsætte. GLP-1 er et nyt koncept til behandling af både glucoseregulation og til potentiel beskyttelse mod storkars-komplikationer. Hypoglycæmi er en hyppig og potentiel alvorlig bivirkning til diabetesbehandling. GLP-1 vil med stor sandsynlighed også kunne udøve beskyttelse af hjernen i forbindelse med hypoglycæmi. Vi mener derfor, at det er berettiget at udføre dette studie for at tilvejebringe nye valide oplysninger om virkningsmekanismen bag GLP-1’s gavnlige virkning i hjerne og hjerte, og at risici og bivirkninger opvejes af de forventede fordele.

Undersøgelsen gennemføres i overensstemmelse med principperne i **Helsinki deklaration II** og vil først blive påbegyndt, når tilladelse fra den lokale Videnskabsetiske komite foreligger.

Skriftlig accept fra de involverede forsøgspersoner skal foreligge før undersøgelsens start.

Forsøgspersoner er forsikringsmæssigt dækket i henhold til ”Bekendtgørelse af lov om patientforsikring” og ” Lov om erstatning for lægemiddelskader”.

**Informeret samtykke**

Undersøgelsens deltagere vil blive informeret både skriftligt og mundtligt om formålet med undersøgelsen, procedurer, potentielle risici eller ubehag samt forventet udbytte.

Det understreges at deltagelse er frivillig samt, at forsøgspersonen til en hver tid har ret til at trække sit tilsagn tilbage og træde ud af undersøgelsen.

Mundtlig information gives af projektkoordinerende læge Susanne Lerche. Forsøgspersonen har mulighed for at medbringe en bisidder til samtalen. I forbindelse med samtalen vil der blive udleveret skriftlig information om undersøgelsen samt Forskningsministeriets folder ”Før du beslutter dig…”

Skriftligt samtykke om deltagelse i studiet indhentes fra forsøgsdeltageren efter en betænkningstid. Samtykkeerklæringen underskrives af både forsøgsansvarlig læge og forsøgspersonen og en kopi af den underskrevne erklæring udleveres til deltageren.

**11. Strålingsdosis og samfundsmæssig nytteværdi**

Der gives i dette studie strålingsdosis svarende til IRCP kategori IIb.

T2D er en sygdom, som er kraftigt stigende i incidens, og hvor særligt komplikationer i form af blodpropper i hjerte og hjerne er ansvarlige for stor morbiditet og mortalitet hos denne patientgruppe. Således kan man i løbet af få år forvente en voldsom stigning også i antallet af disse kardiovaskulære komplikationer.

GLP-1 har i flere studier vist sig at have en beskyttende effekt i CNS og i hjertet netop i forbindelse med iskæmisk skade. Virkningsmekanismen bag disse effekter kendes imidlertid ikke.

Potentielt set vil GLP-1 kunne reducere antallet af ovennævnte komplikationer og deres sværhedsgrad – dvs. yde både sekundær og tertiær forebyggelse.

Vores studie vil give væsentlig information omkring virkningsmekanismen bag disse beskyttende effekter og vil være et vigtigt led i udviklingen af indikationsområder for anvendelse af GLP-1.

Det vil således kunne anvendes både til den glykæmiske behandling ved T2D og til forebyggelse af sygdommens komplikationer. Andre potentielle indikationer vil være behandling af Mb.Alzheimer samt iskæmiske hjerte og hjernesygdom opstået uafhængigt af diabetes.

Én GLP-1 agonist er allerede godkendt til behandling af T2D i USA og flere er på vej.

Strålingsdosis findes således på dette grundlag berettiget idet studiet har stor betydning for forebyggelsen af lidelser med store omkostninger for både samfund og patient.

**12. Databehandling**

Kildedata opføres i CRF (case report form) og/eller en database. Data indtastes 2 gange for at undgå fejlindtastning. Ved kildedata forstås originale dokumenter med kliniske fund, observationer eller øvrige aktiviteter, der er nødvendige for at rekonstruere og evaluere et forsøg. Herunder hører blandt andet laboratoriesvar, udskrifter fra apparatur og lignende. Manuelle personidentificerbare data opbevares forsvarligt aflåst således, at uvedkommende ikke kan gøre sig bekendt med indholdet. I databasen identificeres forsøgsdeltagerne med screeningsnummer, randomiseringsnummer og initialer. Deltagernes initialer forefindes kun i databasen i databearbejdningsperioden. Efter fuldførelse af studiet vil deltagernes initialer blive slettet fra databasen for at sikre deres anonymitet. En liste over screeningsnumre, randomiseringsnumre, initialer og personlige data opbevares adskilt fra de øvrige studiedata, således at det vil være muligt at finde tilbage til kildedata. CPR-nummer vil fremgå af en del af papirdata, hvorfor papirdata vil blive arkiveret aflåst når projektet er afsluttet. Data vil blive opbevaret i 15 år efter studiets afslutning. Projektet anmeldes til Datatilsynet.

**13. Økonomiske forhold**

Til dækning af transportudgifter, tabt arbejdsfortjeneste og som ulempegodtgørelse betales hver forsøgsdeltager 1200,- kr. pr. undersøgelsesdag (besøg 2, 3), i alt 3000,- kr. Beløbet er skattepligtigt.

Den lægelige arbejdskraft udføres af ansøger indenfor eksisterende rammer i forbindelse med ansættelse som Klinisk assistent ved Klinisk Farmakologisk Institut, Århus Universitet og afd. M Århus Sygehus NBG.

Der er modtaget støtte til projektet fra Novonordisk fonden på 150.000 kr., derudover fra Diabetesforeningen på 200.000 kr. og fra Lundbeckfonden på 1.6 millioner kr. (pengene er ikke udelukkende til dette projekt men også til 2 andre projekter)

**14. Tidsperspektiv**

Forventet inklusionsstart i efterår 2006 og afslutning Forår 2007

**15. Publikationspolitik**

Studiet på CNS vil udmunde i en videnskabelig artikel, som publiceres i et internationalt tidsskrift. Medforfattere er studiegruppen med Susanne Lerche som førsteforfatter og Ole E. Schmitz som sidste forfatter.

Studiet på CNS vil blive anvendt som del af et PhD-projekt med titlen *”Potentielle nye virkningsmekanismer af GLP-1,med specielt fokus på CNS og hjerte”*

Studiet på hjertet udmunder i en artikel med HE Bøtker som førsteforfatter, Susanne Lerche som anden forfatter og Ole E. Schmitz som sidste forfatter.

Såvel positive som negative resultater offentliggøres.

**16. Forsøgsansvarlig og studiegruppe**.

**Klinisk ansvarlig investigator og afdelingens forskningsansvarlige professor:**

Ole E. Schmitz, Professor, dr. med. (Klinisk ansvarlig)

Klinisk Farmakologisk Center og Medicinsk Afd. M,

Aarhus Universitetshospital.

- **Ole E. Schmitz er forsøgsansvarlig.**

**Projekt-koordinator:**

Susanne Lerche, Læge, Klinisk assistent,

Farmakologisk Institut, Århus Universitet samt

Forskningsenheden Med. Afd. M

Aarhus Universitetshospital

**Ansvarlig investigator for PET-scanning**

Albert Gjedde, Professor, dr.med

PET-centeret

Aarhus Universitetshospital

**Kardiologisk ansvarlig investigator**

Hans Erik Bøtker, overlæge, dr. med., Ph.D,

Hjertemedicinsk afdeling B, Skejby sygehus

Aarhus Universitetshospital.

Reference List

1. Intensive blood-glucose control with sulphonylureas or insulin compared with conventional treatment and risk of complications in patients with type 2 diabetes (UKPDS 33). UK Prospective Diabetes Study (UKPDS) Group. *Lancet* 352:837-853, 1998

2. The Diabetes Control and Complications Trial Research Group: The Effect of Intensive Treatment of Diabetes on the Development and Progression of Long-Term Complications in Insulin-Dependent Diabetes Mellitus. *N Engl J Med* 329:977-986, 1993

3. DeFronzo RA: Pathogenesis of type 2 diabetes mellitus. *Med.Clin.North Am.* 88:787-835, ix, 2004

4. Evans ML, McCrimmon RJ, Flanagan DE, Keshavarz T, Fan X, McNay EC, Jacob RJ, Sherwin RS: Hypothalamic ATP-sensitive K + channels play a key role in sensing hypoglycemia and triggering counterregulatory epinephrine and glucagon responses. *Diabetes* 53:2542-2551, 2004

5. Blomqvist G, Gjedde A, Gutniak M, Grill V, Widen L, Stone-Elander S, Hellstrand E: Facilitated transport of glucose from blood to brain in man and the effect of moderate hypoglycaemia on cerebral glucose utilization. *Eur.J.Nucl.Med.* 18:834-837, 1991

6. Holst JJ: On the Physiology of GIP and GLP-1. *Horm.Metab Res.* 36:747-754, 2004

7. Nauck MA: Glucagon-like Peptide 1 (GLP-1) in the Treatment of Diabetes. *Horm.Metab Res.* 36:852-858, 2004

8. Degn KB, Brock B, Juhl CB, Djurhuus CB, Grubert J, Kim D, Han J, Taylor K, Fineman M, Schmitz O: Effect of intravenous infusion of exenatide (synthetic exendin-4) on glucose-dependent insulin secretion and counterregulation during hypoglycemia. *Diabetes* 53:2397-2403, 2004

9. Nauck MA, Heimesaat MM, Behle K, Holst JJ, Nauck MS, Ritzel R, Hufner M, Schmiegel WH: Effects of glucagon-like peptide 1 on counterregulatory hormone responses, cognitive functions, and insulin secretion during hyperinsulinemic, stepped hypoglycemic clamp experiments in healthy volunteers. *J.Clin.Endocrinol.Metab* 87:1239-1246, 2002

10. Wei Y, Mojsov S: Tissue-specific expression of the human receptor for glucagon-like peptide-I: brain, heart and pancreatic forms have the same deduced amino acid sequences. *FEBS Lett.* 358:219-224, 1995

11. Steenbergen C, Tong H, Murphy E: PI3 Kinase and Cardioprotection. *Cardiovasc.J S.Afr.* 15:S4, 2004

12. Perry T, Greig NH: The glucagon-like peptides: a new genre in therapeutic targets for intervention in Alzheimer's disease. *J.Alzheimers.Dis.* 4:487-496, 2002

13. Ahren B: GLP-1 and Extra-islet Effects. *Horm.Metab Res.* 36:842-845, 2004

14. During MJ, Cao L, Zuzga DS, Francis JS, Fitzsimons HL, Jiao X, Bland RJ, Klugmann M, Banks WA, Drucker DJ, Haile CN: Glucagon-like peptide-1 receptor is involved in learning and neuroprotection. *Nat.Med.* 9:1173-1179, 2003

15. Perry T, Haughey NJ, Mattson MP, Egan JM, Greig NH: Protection and reversal of excitotoxic neuronal damage by glucagon-like peptide-1 and exendin-4. *J.Pharmacol.Exp.Ther.* 302:881-888, 2002

16. Perry T, Lahiri DK, Chen D, Zhou J, Shaw KT, Egan JM, Greig NH: A novel neurotrophic property of glucagon-like peptide 1: a promoter of nerve growth factor-mediated differentiation in PC12 cells. *J.Pharmacol.Exp.Ther.* 300:958-966, 2002

17. Nikolaidis LA, Mankad S, Sokos GG, Miske G, Shah A, Elahi D, Shannon RP: Effects of glucagon-like peptide-1 in patients with acute myocardial infarction and left ventricular dysfunction after successful reperfusion. *Circulation* 109:962-965, 2004

18. Bose AK, Mocanu MM, Carr RD, Brand CL, Yellon DM: Glucagon-like Peptide 1 Can Directly Protect the Heart Against Ischemia/Reperfusion Injury. *Diabetes* 54:146-151, 2005

19. Nystrom T, Gutniak MK, Zhang Q, Zhang F, Holst JJ, Ahren B, Sjoholm A: Effects of glucagon-like peptide-1 on endothelial function in type 2 diabetes patients with stable coronary artery disease. *Am.J.Physiol Endocrinol.Metab* 287:E1209-E1215, 2004

20. Nielsen MF, Caumo A, Chandramouli V, Schumann WC, Cobelli C, Landau BR, Vilstrup H, Rizza RA, Schmitz O: Impaired basal glucose effectiveness but unaltered fasting glucose release and gluconeogenesis during short-term hypercortisolemia in healthy subjects. *Am.J Physiol Endocrinol.Metab* 286:E102-E110, 2004

21. Botker HE, Wiggers H, Bottcher M, Christiansen JS, Nielsen TT, Gjedde A, Schmitz O: Short-term effects of growth hormone on myocardial glucose uptake in healthy humans. *Am.J.Physiol Endocrinol.Metab* 278:E1053-E1059, 2000

22. Toft-Nielsen MB, Madsbad S, Holst JJ: Continuous subcutaneous infusion of glucagon-like peptide 1 lowers plasma glucose and reduces appetite in type 2 diabetic patients. *Diabetes Care* 22:1137-1143, 1999

23. Botker HE, Bottcher M, Schmitz O, Gee A, Hansen SB, Cold GE, Nielsen TT, Gjedde A: Glucose uptake and lumped constant variability in normal human hearts determined with [18F]fluorodeoxyglucose. *J.Nucl.Cardiol.* 4:125-132, 1997

24. Naslund E, Bogefors J, Skogar S, Gryback P, Jacobsson H, Holst JJ, Hellstrom PM: GLP-1 slows solid gastric emptying and inhibits insulin, glucagon, and PYY release in humans. *Am.J.Physiol* 277:R910-R916, 1999

Effekten af GLP-1 på glucoseoptagelsen i CNS og hjertet hos raske personer under *hypoglycæmi* vurderet ved Positron Emissions Tomografi

**Samtykkeerklæring**

_______________________________________

Forsøgspersonens navn

Jeg bekræfter hermed, at jeg, efter at have modtaget vedlagte information såvel mundtligt som skriftligt, indvilger i at deltage i det beskrevne forsøg.

Jeg er informeret om, at det er frivilligt at deltage samt, at jeg når som helst og uden angivelse af årsag kan trække mit tilsagn tilbage.

Jeg er informeret om at der gemmes blod til eventuelle senere analyser.

_______________________________________

Forsøgspersonens underskrift Dato

Informerende læge(Blokbogstaver)

_______________________________________

Informerende læges underskrift Dato

**Information til forsøgspersoner**

Effekten af GLP-1 på glucoseoptagelsen i CNS og hjertet hos raske personer under *hypoglycæmi* vurderet ved Positron Emissions Tomografi

Vi vil anmode Dem om at deltage i et videnskabeligt forsøg, hvor vi ønsker at undersøge effekten af hormonet glucagon-like-peptid-1(**GLP-1)** på optagelsen af sukker i hjernen og i hjertet. Undersøgelsen foregår på raske mænd mellem 20 og 50 år. Kvinder undersøges i første omgang ikke idet deres insulinfølsomhed varierer i takt med menstruationscyklus.

Type 2 sukkersyge er en sygdom med talrige komplikationer. Særligt en 3 x forøget risiko for blodpropper i hjerte og hjerne i forhold til normalbefolkningen er med til at gøre sygeligheden og dødeligheden ved denne sygdom høj. Ingen af de medikamenter som i dag anvendes i behandlingen af type 2 sukkersyge nedsætter risikoen for udvikling af disse komplikationer.

Lavt blodsukker er en hyppig bivirkning til diabetesbehandling og kan især give skader på hjernen.

GLP-1 er et naturligt forekommende hormon der hos alle mennesker produceres i celler i tarmen som respons på fødeindtagelse. Det stimulerer særligt produktionen af insulin fra bugspytkirtlen og medvirker derfor til at optage og deponere det sukker, som indtages i forbindelse med et måltid. Derudover er hormonet vist at stimulere nydannelsen af insulinproducerende celler i bugspytkirtlen. Hos mennesker med type 2 diabetes er produktionen af GLP-1 nedsat.

GLP-1 behandling forbedrer sukkerstofskiftet væsentligt hos diabetespatienter.

Nyere forskning på dyr har vist, at GLP-1 har evnen til at beskytte hjerneceller mod beskadigelse og celledød. Ligeledes er GLP-1 på mennesker vist at beskytte hjertemusklen i forbindelse med blodprop i hjertet således at skaden på hjertet mindskes.

Således kan GLP-1 potentielt reducere den skade som sker i forbindelse med en blodprop i hjerte eller hjerne og muligvis til en vis grad forebygge deres opståen. Ligeledes er det sandsynligt at GLP-1 vil kunne udøve beskyttelse af hjernen i forbindelse med lavt blodsukker.

Det er fortsat uvist via hvilke mekanismer GLP-1 udøver sin potentielle cellebeskyttelse og der er derfor behov for undersøgelser til at belyse dette og derved øge forståelsen for mekanismerne bag virkningen. I vores studie anvender vi en ny metode til at påvise en mulig virkningsmekanisme for GLP-1´s beskyttelse i hjerne og hjerte i forbindelse med lavt blodsukker.

Undersøgelsen vil blive foretaget på 10 raske mandlige forsøgspersoner. Alle personer skal gennemgå undersøgelse 2 gange henholdsvis **med og uden GLP-1** tilførsel efter tilfældig udvælgelse.

Forsøget består af **4 besøgsdage:**

**1. dag** er et screeningsbesøg, hvor Deres egnethed til at deltage bekræftes ved blodprøver og almindelig fysisk undersøgelse, dette besøg varer ca. ½-1 time.

**2.og 3. dag** er PET-scanningsdage, hvert af disse besøg varer ca. 8-10 timer. Der vil være ca. 4 uger imellem 2. og 3. undersøgelsesdag.

Hjernens og hjertets sukkerstofskifte måles ved hjælp af Positron Emissions Tomografi (PET). Man måler sukkeroptagelse i hjernen og i hjertet ved scanning udefra v.hj.a. naturligt forekommende sporstoffer, der er mærket med ganske små mængder radioaktivitet.

Selve PET skanningen er uden ubehag. Der er ingen lydafgivelse fra scanneren fraset en svag blæselyd. Undersøgelsen indbefatter indsprøjtning af et radioaktivt sporstof i blodbanen og efterfølgende måling af fordelingen i hjernen og i hjertet.

For at undgå påvirkninger af sukkerstofskiftet fra andre hormonsystemer i kroppen sættes kroppen i en basaltilstand ved hjælp af tilførsel af forskellige naturlige hormoner. Blodsukkeret vil under undersøgelsen blive sænket langsomt og til sidst ende på et niveau under det blodsukker man normalt har ved faste. Ved det laveste blodsukker-niveau vil PET-scanningen blive foretaget.

**4. dag** vil der blive foretaget en MR-scanning for at kortlægge hjernens anatomi. Scanningen varer 5-10 min og er uden ubehag.

De vil få såvel skriftlig som mundtlig information om undersøgelsens formål, forløb og risici.

Det understreges, at De når som helst kan trække Dem ud af undersøgelsen.

**På undersøgelsesdagen**

Før undersøgelsen skal De faste fra kl. 22 aftenen inden, dog må De gerne indtage vand, men ikke kaffe, te eller juice.

Der anlægges 3 tynde plastkanyler(venflon) i armene, en i hver albuebøjning og en på underarm/hånd. Hånden vil blive placeret i opvarmet plastbeholder.

Gennem disse kanyler gives sporstoffet, der anvendes til PET-scanningen og ligeledes gives de kontrollerede mængder hormoner i form af insulin, væksthormon, glucagon og somatostatin samt sukkervand for at opretholde basale niveauer af både hormoner og sukker. Der gives ligeledes

GLP-1 infusion gennem en af kanylerne.

Der vil løbende blive taget blodprøver gennem den opvarmede venflon i underarmen. Der vil maximalt udtages 200ml blod under undersøgelsen.

Derudover anlægges en kanyle i en pulsåre ved håndleddet som skal bruges til at måle radioaktivitet. Målingerne anvendes i den senere analyse af PET-scanningsbillederne.

Herefter opstartes hormoninfusioner og sukkerinfusion, som vil fortsætte under hele undersøgelsen. Der vil blive målt blodsukker ca. hver 5.-10 min gennem hele undersøgelsen og derudover ca. hver ½ time flere andre blodprøver.

Efter 60 minutter opstartes GLP-1 infusion eller placebo(saltvand), som skal fortsætte i resten af undersøgelsen. Ca. 4½ time efter undersøgelsesstart placeres De på et leje i PET-scanneren med først hovedet og senere brystkassen i scanneren. Scanner-åbningens dybde er ca. 60cm og den er ca. 80cm i diameter. De understøttes af forskellige puder, så De ligger behageligt. De kan under hele undersøgelsen komme i kontakt med den ansvarlige læge og det øvrige personale.

Efter ca. 4 timers GLP-1 infusion gives sporstoffet FDG, som svarer til naturligt forekommende sukkerforbindelser, der optages i hjernen og i hjertet. Umiddelbart herefter startes PET-scanningsproceduren, som først består af en 25 minutters varende forberedelsesscanning og derefter sukkerscanningen. Scanningen vil ca. vare 2 timer.

Efter endt scanning stoppes alle hormon-infusioner, dog fortsættes sukker-infusionen indtil blodsukkeret er stigende. Blodsukkeret kontrolleres efterfølgende hver 5.-10.minut indtil det er stabilt i normalt niveau. Der serveres en let frokost og De udstyres med druesukker inden De forlader undersøgelsesstedet.

**Risici og ulemper**

**Hormoner**

De vil sandsynligvis opleve symptomer på lavt blodsukker under sidste del af undersøgelsen. Disse kan være træthed, sultfornemmelse, rysten i kroppen samt alment ubehag.

GLP-1 infusion kan hos nogle personer give let kvalme, ubehag i maven eller hovedpine.

Somatostatin kan give anledning til kvalme, opkastninger og mavesmerter.

Alle hormoner der i øvrigt indgives er naturligt forekommende i organismen og gives udelukkende i normale fysiologiske doser.

**PET-undersøgelsen**

Før skanningen skal De have lagt en plastikkanyle (venflon) i en blodåre på underarmen og i begge albuebøjninger. Dette kan medføre et blåt mærke eller rødme af huden. I sjældne tilfælde kan der opstå betændelse i huden. Hvis der kommer tegn på betændelse(rødme, varme, ømhed, evt. feber), skal De henvende Dem til en af lægerne, der står for projektet.

Kanylen i pulsåren anlægges af en erfaren speciallæge i anæstesi. I meget sjældne tilfælde kan ses komplikationer i form af blødning, betændelse, seneskade og blodprop i armen.

En PET-undersøgelse indbefatter indsprøjtning af et sporstof, som indeholder radioaktivitet.

Den totale stråledosis De modtager svarer til 3 gange den årlige baggrundsstråling, som alle i Danmark udsættes for. Man kan teoretisk beregne, at den samlede risiko for på længere sigt at dø af kræft øges med 0.04%. Risikoen øges således fra 25,0 (alle menneskers risiko for at udvikle kræft gennem livet) til 25,04 %, dette dog set i lyset af, at undersøgelsen giver **væsentlig** information vedrørende GLP-1’s anvendelse i forebyggelsen af blodpropper i hjerne og hjerte hos patienter både med og uden type 2 diabetes patienter. Ligeledes vedrørende GLP-1’s mulige plads i behandlingen af patienter med Alzheimers demens.

**MR-scanning**

MR-scanning anses for at være en sikker undersøgelse uden risici. Scanneråbningen er lille hvilket kan give anledning til klaustrofobiske gener.

Undersøgelsens oplysninger vil kun være tilgængelige for medarbejderne, forsøgspersonerne og for revision fra offentlige myndigheder, hvilket er i overensstemmelse med dansk registerlovgivning.

Undersøgelsen er godkendt af Videnskabsetisk Komite for Århus Amt før start.

Undersøgelsen vil udmunde i to videnskabelige artikler, som udgives i et større internationalt tidsskrift.

Der vil blive gemt blod til senere analyse af stress-respons i cellerne. Analysen er ikke tilgængelig endnu. Såfremt analysen umuliggøres vil det gemte blod blive destrueret. Blodet vil ikke blive anvendt til andet.

**Frivillighed**

Deltagelse i undersøgelsen er frivillig. Man kan til enhver tid og uden begrundelse, trække et givet tilsagn tilbage uden, at det vil påvirke eventuel fremtidig behandling.

De har ret til betænkningstid før samtykke afgives, ligesom De såfremt De ønsker det, kan medbringe en bisidder ved modtagelsen af den mundtlige information.

Vedlagt er folderen ”Før du beslutter dig” fra Forskningsministeriet.

Undersøgelsen er et videnskabeligt projekt, som den enkelte deltager ikke selv får gavn af, men har til formål at hjælpe til forståelsen af, hvorledes GLP-1 udøver sin gavnlige virkning i hjernen og hjertet.

**Udtrædelse af forsøget**

De kan således til en hver tid trække dem ud af studiet. For Deres vedkommende vil forsøget ligeledes afbrydes såfremt der opstår alvorlige bivirkninger eller bivirkninger som efter den forsøgsansvarlige læges vurdering nødvendiggør udtrædelse. Derudover, hvis De ikke kan opfylde kravene til gennemførelse af studiet.

**Forsikring**

De er dækket af, dels Patientforsikringen, dels Lægemiddelforsikringen, hvis der skulle ske påvirkninger af Deres helbredstilstand som følge af deltagelse i undersøgelsen.

Projektet finansieres med økonomisk støtte fra offentlige fonde. Aktuelt er der opnået støtte fra NovoNordisk Fonden, Diabetesforeningen og Lundbeckfonden.

Der udbetales en ulempegodtgørelse på 3000 kr. for deltagelse i hele forsøget. Beløbet er skattepligtigt. Derudover kan der godtgøres transportudgifter.

**Projektgruppen** består af følgende personer, som frit kan kontaktes vedrørende spørgsmål om undersøgelsen:

Susanne Lerche, Læge, Klinisk assistent.

Medicinsk Forskningsafsnit M, Århus Sygehus NBG,

Tlf. 89492071 eller 26111789. E-mail: lerche@ki.au.dk

Ole E. Schmitz, Professor, overlæge, dr.med.,

Farmakologisk Institut, Århus Universitet og Medicinsk afd. M, Århus Sygehus NBG,

Tlf. 8949-4444

Hans Erik Bøtker, Overlæge, dr. med., Ph.D,

Hjertemedicinsk afdeling B, Skejby sygehus,

Tlf. 8949-5566

Albert Gjedde, Professor, dr.med

PET-centeret, Århus Sygehus NBG,

Tlf. 8949-4444

**Lægmandsbeskrivelse**

**Effekten af GLP-1 på glucoseoptagelsen i CNS og hjertet hos raske personer under *normoglycæmi* vurderet ved Positron Emissions Tomografi**

Type 2 sukkersyge er en sygdom med talrige komplikationer. Særligt en 3 x forøget risiko for blodpropper i hjerte og hjerne i forhold til normalbefolkningen er med til at gøre sygeligheden og dødeligheden ved denne sygdom høj. Ingen af de medikamenter, som i dag anvendes i behandlingen af type 2 sukkersyge nedsætter risikoen for udvikling af disse komplikationer.

Lavt blodsukker er en hyppig bivirkning til diabetesbehandling og kan især give skader på hjernen.

Glucagon-like-peptid-1(**GLP-1**) er et naturligt forekommende hormon der hos alle mennesker produceres i celler i tarmen og frigives som svar på fødeindtagelse. Det stimulerer særligt produktionen af insulin fra bugspytkirtlen og medvirker derfor til at optage og deponere det sukker, som indtages i forbindelse med et måltid. Derudover er hormonet vist at stimulere nydannelsen af insulinproducerende celler i bugspytkirtlen.

Hos mennesker med type 2 diabetes er produktionen af GLP-1 nedsat.

GLP-1 behandling forbedrer sukkerstofskiftet væsentligt hos sukkersygepatienter.

Nyere forskning på dyr har vist, at GLP-1 har evnen til at beskytte hjerneceller mod beskadigelse og celledød. Ligeledes er GLP-1 på mennesker vist at beskytte hjertemusklen i forbindelse med blodprop i hjertet, så skaden på hjertet mindskes.

Således kan GLP-1 potentielt reducere den skade, som sker i forbindelse med en blodprop i hjerte eller hjerne og muligvis til en vis grad forebygge deres opståen.

Ligeledes er det meget sandsynligt, at GLP-1 vil kunne udøve beskyttelse af hjernen og hjertet i forbindelse med lavt blodsukker.

Det er fortsat uvist via hvilke mekanismer GLP-1 udøver denne potentielle cellebeskyttelse og der er derfor behov for undersøgelser til at belyse dette og derved øge forståelsen for mekanismerne bag virkningen. I dette studie anvendes en ny metode til at påvise en mulig virkningsmekanisme for GLP-1´s beskyttelse i hjerne og hjerte.

Undersøgelsen foretages på 10 raske mandlige forsøgspersoner. Alle personer skal gennemgå undersøgelse 2 gange henholdsvis **med og uden GLP-1** tilførsel.

Forsøget består af 1 forundersøgelse varende ca. ½-1 time og af 2 undersøgelsesdage, som hver varer ca. 8-10 timer.

Hjernens og hjertets sukkerstofskifte måles ved hjælp af Positron Emissions Tomografi som ikke kræver direkte adgang til hverken hjertet eller hjernen. For at undgå påvirkninger af sukkerstofskiftet fra andre hormonsystemer i kroppen sættes kroppen i en basaltilstand ved hjælp af tilførsel af forskellige naturligt forekommende hormoner. Samtidig vil blodsukkeret under undersøgelsen blive sænket langsomt og ende i et niveau under det blodsukker man normalt har under faste. PET-scanningen foretages ved det laveste blodsukker.

Metoden kaldes ”Pancreatisk clamp” og har været anvendt i Diabetesafdelingen talrige gange uden komplikationer.Samtidig med den pancreatiske clamp gives infusion med GLP-1 eller saltvand efter tilfældig udvælgelse.

Selve PET skanningen er uden ubehag. Den indbefatter indsprøjtning af radioaktive sporstoffer i blodbanen og efter følgende måling af deres fordeling i hjernen og i hjertet.

Proceduren anvendes rutinemæssigt i udredningen af kardiologiske og neurologiske lidelser.

Den totale stråledosis svarer til 3 gange den årlige baggrundsstråling, som alle mennesker i Danmark udsættes for. Man kan teoretisk beregne, at den samlede risiko for på længere sigt at dø af kræft øges med 0.06%. Risikoen øges således fra 25,0 (alle menneskers risiko for at udvikle kræft gennem livet) til 25,06 %.

Forsøgspersonerne gives såvel skriftlig som mundtlig information om undersøgelsens formål, forløb og risici. Det understreges, at forsøgspersonerne når som helst, og uden begrundelse kan trække sig ud af undersøgelsen, og at det i givet fald ikke vil få indflydelse på en eventuel fremtidig behandling i afdelingen.

Der gives økonomisk kompensation til forsøgsdeltagerne til dækning af transport, tabt arbejdsfortjeneste og ulejlighed. Beløbet er på 3000kr og er skattepligtigt.

Projektet finansieres med økonomisk støtte fra offentlige fonde.

**Fuldmagt**

**Effekten af GLP-1 på glucoseoptagelsen i CNS og hjertet hos raske personer under *hypoglycæmi* vurderet ved Positron Emissions Tomografi**

**Jeg giver fuldmagt til at oplysninger fra min patientjournal (jf. lov om patienters retsstilling §24) kan blive set af Den Videnskabsetiske Komité, Lægemiddelstyrelsen samt Datatilsynet eller tilsvarende myndighed i indtil 15 år efter undersøgelsens afslutning. Dette sker i henhold til §20 i ”Lov om patienters retsstilling”.**

**Denne fuldmagt gælder kun oplysninger, som har relation til denne undersøgelse, og den kan til enhver tid tilbagekaldes. Sådanne oplysninger behandles fortroligt.**

Navn (blokbogstaver)

Underskrift Dato

| **Forsøgsdeltagere søges til forskningsprojekt** |
| --- |

**Du kan deltage i forsøget, hvis du:**

- Er mand
- Er mellem 20 og 50 år
- Er normalvægtig
- Er ikke-ryger
- Ikke har nære familiemedlemmer med sukkersyge

**Om forsøget:**

- Formålet med forsøget er med ny metodologi at undersøge virkningen af hormonet **GLP-1** på sukker-optagelsen i hjerne og hjerte uafhængigt af insulin **under hypoglycæmi** (lavt blodsukker)
- Undersøgelsen består af 1 kort screeningsbesøg , en kort MR-scanning og 2 undersøgelsesdage (4 uger imellem), som hver varer ca. 8-10 timer. Du skal være fastende og sengeliggende under hele undersøgelsen og må ikke sove.
- Sukkeroptagelsen måles ved hjælp af **PET-scanning**
- Kroppen sættes i en ”basaltilstand” ved hjælp af tilførsel af forskellige naturligt forekommende hormoner.
- Blodsukkeret sænkes langsomt til under normalt faste-niveau, hvorefter scanningen foretages.
- En udførlig forsøgsbeskrivelse vil blive udleveret ved henvendelse til undertegnede.
- Forsøget er godkendt af Den Videnskabsetiske Komite i Århus Amt
- Der vil blive ydet en økonomisk kompensation på i alt 3000kr for deltagelse i hele forskningsprojektet.

**Er du interesseret kan yderligere information rekvireres hos:**

Susanne Lerche, læge, klinisk assistent

Forskningsenheden, Med. Afd. M, Århus Sygehus NBG

e-mail: lerche@ki.au.dk

Tlf.: 26111789 eller 89492071,
